# Recent Progress of Electrochemical Production of Hydrogen Peroxide by Two‐Electron Oxygen Reduction Reaction

**DOI:** 10.1002/advs.202100076

**Published:** 2021-05-27

**Authors:** Nan Wang, Shaobo Ma, Pengjian Zuo, Jizhou Duan, Baorong Hou

**Affiliations:** ^1^ Key Laboratory of Marine Environmental Corrosion and Bio‐Fouling Institute of Oceanology Chinese Academy of Sciences 7 Nanhai Road Qingdao 266071 China; ^2^ Center for Ocean Mega‐Science Chinese Academy of Sciences 7 Nanhai Road Qingdao 266071 China; ^3^ Open Studio for Marine Corrosion and Protection Pilot National Laboratory for Marine Science and Technology (Qingdao) 1 Wenhai Road Qingdao 266237 China; ^4^ MITT Key Laboratory of Critical Materials Technology for New Energy Conversion and Storage School of Chemistry and Chemical Engineering Harbin Institute of Technology Harbin 150001 China

**Keywords:** application, catalytic mechanism, electrochemical catalysts, hydrogen peroxide, two‐electron oxygen reduction reaction

## Abstract

Shifting electrochemical oxygen reduction reaction (ORR) via two‐electron pathway becomes increasingly crucial as an alternative/green method for hydrogen peroxide (H_2_O_2_) generation. Here, the development of 2e^−^ ORR catalysts in recent years is reviewed, in aspects of reaction mechanism exploration, types of high‐performance catalysts, factors to influence catalytic performance, and potential applications of 2e^−^ ORR. Based on the previous theoretical and experimental studies, the underlying 2e^−^ ORR catalytic mechanism is firstly unveiled, in aspect of reaction pathway, thermodynamic free energy diagram, limiting potential, and volcano plots. Then, various types of efficient catalysts for producing H_2_O_2_ via 2e^−^ ORR pathway are summarized. Additionally, the catalytic active sites and factors to influence catalysts’ performance, such as electronic structure, carbon defect, functional groups (O, N, B, S, F etc.), synergistic effect, and others (pH, pore structure, steric hindrance effect, etc.) are discussed. The H_2_O_2_ electrogeneration via 2e^−^ ORR also has various potential applications in wastewater treatment, disinfection, organics degradation, and energy storage. Finally, potential future directions and prospects in 2e^−^ ORR catalysts for electrochemically producing H_2_O_2_ are examined. These insights may help develop highly active/selective 2e^−^ ORR catalysts and shape the potential application of this electrochemical H_2_O_2_ producing method.

## Graphical abstract

Electrochemical oxygen reduction reaction (ORR) via 2e^−^ pathway becomes increasingly crucial for hydrogen peroxide (H_2_O_2_) generation. This work comprehensively reviews the recent works of 2e^−^ ORR catalysts, in aspects of catalytic mechanism, types of catalysts, factors to influence catalytic performance and potential applications. Additionally, potential strategies are given for developing high‐performance 2e^−^ ORR catalysts and on‐site applications.

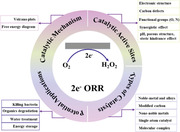

## 1 Introduction

H_2_O_2_ is a valuable oxidative chemical with rapidly growing demand in various applications, including the chemical synthesis, pulp/paper bleaching, and wastewater treatment (organic pollutants degradation/drinking water purification).^[^ ^1^ ^]^ The current industrial scale synthesis of H_2_O_2_ involves an energy‐intensive anthraquinone oxidation–reduction.^[^ ^2^ ^]^ However, this multistep synthesis method generally requires complex large‐scale infrastructure and expensive palladium hydrogenation catalysts, which also generates a substantial volume of organic byproduct wastes. The direct synthesis of H_2_O_2_ from hydrogen and oxygen provides a more straightforward and atom‐economic process, to ideally solve the issues associated with the complex anthraquinone route.^[^ ^3^ ^]^ Nevertheless, this direct synthesis method generally needs the use of platinum‐group noble‐metal catalysts with low catalytic efficiency and faces the potential explosion hazard of oxygen/hydrogen mixtures, which make its commercial application doubtful. Another attractive and alternative route for the on‐site direct production of H_2_O_2_ is an electrochemical process, which can ideally solve the issues associated with the indirect anthraquinone route and direct synthesis of H_2_O_2_ from H_2_ and O_2_.^[^ ^4^ ^]^ The electrochemical production of H_2_O_2_ can be realized mainly through two pathways, including the 2e^−^ oxygen reduction and water oxidation. In this paper, the H_2_O_2_ generation via 2e^−^ oxygen reduction pathway will be discussed in detail.

Molecular oxygen generally can be electrochemically reduced to H_2_O via a 4e^−^ transferred pathway, or H_2_O_2_ with 2e^−^ pathway in aqueous solution.^[^ ^5^ ^]^ Substantial efforts in recent years have aimed at efficiently generating electricity simultaneously with a high‐yield production of H_2_O_2_. In the 1930s, Berl firstly reported to produce H_2_O_2_ through electrochemical reduction of oxygen, which was further adopted to produce dilute alkaline H_2_O_2_ via the well‐known Huron–Dow process.^[^ ^6^ ^]^ The Huron–Dow process was commercialized in 1991, due to that the produced dilute alkaline H_2_O_2_ could be directly used for pulp and paper bleaching process.^[^ ^6^ ^]^ However, the Huron–Dow process can only produce low‐purity and high alkalinity H_2_O_2_ solution product, which not work in acid and neutral solution. Recently, the electro‐Fenton process, based on a mixture of electrochemical produced H_2_O_2_ and Fe^2+^ ion, is widely studied to produce hydroxyl radicals (·OH), which can be further used to degrade organic pollutants.^[^ ^1a^ ^]^ Microbial electrochemical cell, constructed with microbes containing anode and acetate (waste‐water) electrolyte, can also produce H_2_O_2_ at cathode side, while it always exhibits a lower catalytic efficiency.^[^ ^7^ ^]^ For the 2e^−^ ORR route to generate H_2_O_2_, exploring electrocatalysts with high activity and selectivity in acid/neutral/alkaline electrolyte is prerequisite. Until now, a variety of materials are investigated as 2e^−^ ORR catalysts, such as noble metal/alloys, functional (O, N, F, S, or B doped) carbon materials, non‐noble transition metals, single‐atom catalysts (SACs) and molecular complexes.^[^ ^8^ ^]^ However, the reported catalysts still face the unsatisfactory catalytic performances. There is still a long way to realize the large‐scale production of H_2_O_2_ via electrochemical 2e^−^ ORR pathway. The critical knob for exploring efficient catalysts relies on the proper binding strength between the reaction catalytic sites and oxygen/oxygen transition species. A too‐strong interaction may easily dissociate the O_2_ molecule toward H_2_O via 4e^−^ pathway, while a too‐weak interaction may cause a high selectivity to H_2_O_2_ but lower catalytic activity.^[^ ^5a^ ^]^ Therefore, an ideal 2e^−^ ORR electrocatalyst with flexible tenability in electronic structures is highly desired for systematic control of 2e^−^ ORR pathways as well as improvements in catalytic activities.

**Figure 1:**
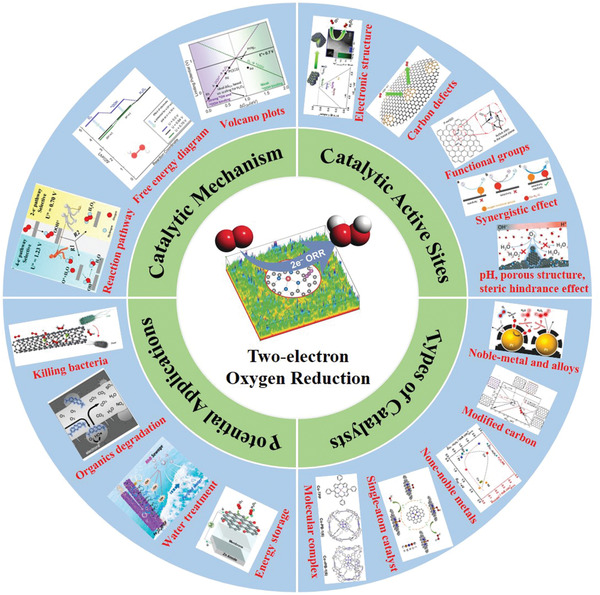
Schematic diagram of 2e^−^ ORR electrochemical catalysts summarized in this review, including catalytic mechanism, catalytic active sites, types of catalysts and potential applications. Reaction pathway: Reproduced with permission.^[^ ^12^ ^]^ Copyright 2019, American Chemical Society. Free engery diagram and volcano plots: Reproduced with permission.^[^ ^5a^ ^]^ Copyright 2018, American Chemical Society. Electonic structure: Reproduced with permission.^[9a^ ^]^ Copyright 2014, American Chemical Society. Carbon defects: Reproduced with permission.^[^ ^43^ ^]^ Copyright 2018, American Chemical Society. Functional groups: Reproduced with permission.^[^ ^55^ ^]^ Copyright 2018, Springer Nature. Synergistic effect: Reproduced with permission.^[^ ^65^ ^]^ Copyright 2019, Wiley‐VCH. Porous structure: Reproduced with permission.^[^ ^66^ ^]^ Copyright 2014, American Chemical Society. Noble‐metal and alloys: Reproduced with permission.^[^ ^23a^ ^]^ Copyright 2014, American Chemical Society. Modified carbon: Reproduced with permission.^[^ ^40a^ ^]^ Copyright 2018, American Chemical Society. None‐noble metals: Reproduced with permission.^[16^ ^]^ Copyright 2019, American Chemical Society. Single‐atom catalyst: Reproduced with permission.^[^ ^17^ ^]^ Copyright 2020, American Chemical Society. Molecular complex: Reproduced with permission.^[^ ^95^ ^]^ Copyright 2020, Wiley‐VCH. Energy storage: Reproduced with permission.^[^ ^88^ ^]^ Copyright 2019, Springer Nature. Water treatment: Reproduced with permission.^[^ ^78^ ^]^ Copyright 2019, Elsevier. Organics degradation: Reproduced with permission.^[^ ^118^ ^]^ Copyright 2019, American Chemical Society. Killing bacteria: Reproduced with permission.^[122^ ^]^ Copyright 2018, American Chemical Society. Center image: Reproduced with permission.^[^ ^86^ ^]^ Copyright 2020, Wiley‐VCH.

In this review, we summarized the development of two‐electron electrochemical ORR catalysts in recent years, in aspects of catalytic mechanism, types of high‐performance catalysts, factors to influence catalytic performance and potential applications (**Figure** **1**). Until now, a diverse range of electrochemical catalysts are investigated for the electrochemical synthesis of H_2_O_2_, such as noble metal/alloys, modified carbon materials, non‐noble transition metals, single‐atom catalysts and molecular complexes. Various factors are elucidated to influence the catalytic performance of H_2_O_2_ electrogeneration, including electronic structure, carbon defect, functional groups (O, N, B, S, F, etc.), synergistic effect, pH, pore structure, and steric hindrance effect. Additionally, the electrochemical synthesis of H_2_O_2_ exhibits attractive potential applications, containing disinfection, organics degradation, wastewater treatment, and energy storage. Based on the current researches and crucial challenges of 2e^−^ ORR, we further proposed the further development of 2e^−^ ORR, including mechanism exploring, rational design of catalysts, catalytic activity optimization in various electrolytes and potential applications.

## 2 Catalytic Mechanism of 2e^−^ ORR to H_2_O_2_

### 2.1 Reaction Pathway and Free Energy Diagram

The electrochemical oxygen reduction reaction always includes two kinds of reaction pathways. The first one is 4e^−^ associative reaction pathway, as shown in Equation (1), which involves four primitive steps and three different reaction intermediates, namely *OOH, *O, and *OH, respectively. The second one is 2e^−^ associative pathway, corresponding to partial reduction of oxygen to H_2_O_2_, which contains two primitive steps and only one OOH* reaction intermediate (Equation (2)). The catalytic activity and selectivity of the catalysts toward H_2_O_2_ production are mainly determined by the binding free energy of *OOH intermediate (Δ*G*(*OOH))

(1) $$\begin{array}{ccc} O_{2} & + & 4\left(H^{+}+e^{-}\right)\to *\mathrm{OOH}+3\left(H^{+}+e^{-}\right)\to *O\\ & + & 2\left(H^{+}+e^{-}\right)\to *\mathrm{OH}+1\left(H^{+}+e^{-}\right)\to H_{2}O\;\;E^{0}=\;1.23\;V \end{array}$$

(2) $$\begin{array}{c} O_{2}+2\left(H^{+}+e^{-}\right)\to *\mathrm{OOH}+\left(H^{+}+e^{-}\right)\to H_{2}O_{2}\;E^{0}=0.7\;V \end{array}$$

**Figure 2:**
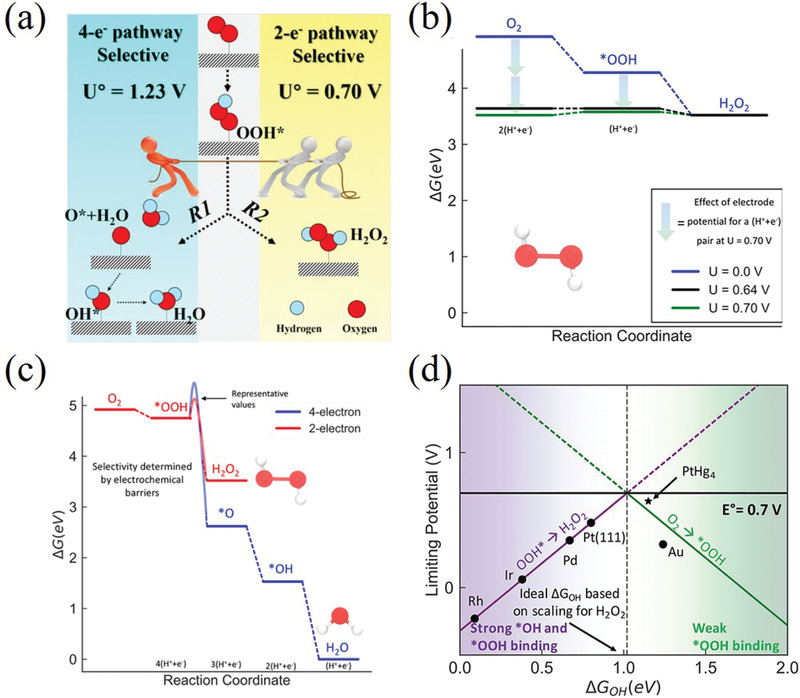
a) Schematic illustration of the reactions and the equilibrium potentials during the oxygen electrochemical reduction. Reproduced with permission.^[^ ^12^ ^]^ Copyright 2019, American Chemical Society. b) Free energy diagram of PtHg_4_ on 2e^−^ ORR at three different potentials: 0 V (blue lines), the corresponding equilibrium potential (green lines), and the limiting potential (black lines). The blue‐green arrows indicate the effect of the potential based on the RHE model. Reproduced with permission.^[^ ^13^ ^]^ Copyright 2013, Springer Nature. c) Free energy diagram for the four‐electron (blue line) and two‐electron (red line) oxygen reduction. The electrochemical barrier for the *OOH to H_2_O_2_ or *O are illustrative and indicate the importance of kinetics in determining catalyst selectivity. d) Limiting potentials for individual steps in Equations (4) and (5), showing the strongly bound *OH region (solid purple line) and weakly bound *OOH region (solid green line) for the 2e^−^ process. The color gradient indicates the strong *OH and weak *OOH binding regions. c,d) Reproduced with permission.^[^ ^5a^ ^]^ Copyright 2018, American Chemical Society.

In the oxygen reduction process, there is an obvious competition between 4e^−^ reaction pathway and 2e^−^ reaction pathway (**Figure** **2**a). Specifically, hydroperoxide species have three possible subsequent pathways, 1) diffuse directly into the electrolyte as final product, 2) further electrochemical reduced to OH^−^ via 4e^−^ pathway, 3) chemical decomposed to O_2_ and OH^−^. As H_2_O is the thermodynamically favored product, it is an obstacle for synthesizing H_2_O_2_ product via 2e^−^ ORR pathway. As shown in Figure 2a, the *OOH is the common intermediate between the two ORR pathways, and the bond strength between catalysts and *OOH intermediate determines the reaction product. Thus, the final product of the ORR process strongly depends on the electrochemical catalysts.

Density functional theory (DFT) calculations work well in describing adsorption energies of intermediates on catalysts surface, except the energy of the solvated protons and the electrons in the electrode at a given potential. Nørskov et al. firstly developed a typical calculation method upon the catalytic process, based on computational hydrogen electrode (CHE) model. The free energy of a single proton−electron pair is defined as −e*U* relative to H_2_ in the gas phase at standard conditions, where *U* is the electrode potential versus the reversible hydrogen electrode (RHE).^[^ ^9^ ^]^ The adsorption free energy of an intermediate (with n proton−electron pairs) generally consists of several parts, including calculated binding energy (Δ*E*_ele_), adsorbate solvation (Δ*E*_w_), electric field effects (Δ*E*_field_), zero‐point energy (Δ_ZPE_) and entropic corrections (−*T*Δ*S*), as displayed in Equation (3)

(3) $$\Delta G\;=\;\Delta E_{\mathrm{ele}}+\Delta E_{w}+\Delta E_{\mathrm{field}}+\Delta_{\mathrm{ZPE}}-T\Delta S-neU$$

Figure 2b shows the DFT‐calculated free energy diagram of Pt–Hg_4_ for the 2e^−^ ORR process using the CHE model. At *U* = 0.0 V (blue lines), all the reaction steps are downhill, indicating it is a facile reaction at this state. The green line represents the thermodynamics free energy of sole *OOH intermediate at the equilibrium potential (*U* = 0.7 V), and the adsorption energies are shifted by −*n*e*U* based on Equation. Figure 2b shows that the reduction of O_2_ to *OOH is uphill in energy at *U* = 0.7 V (green lines), implying this limiting reaction step may hinder further adsorption and dissociation of O_2_. Based on the CHE model, the thermodynamic limiting potential (*U*_L_) is defined as the highest potential, at which all the reaction steps are downhill in free energy. The calculated *U*_L_ of Pt–Hg_4_ catalyst is 0.64 V, and free energy diagram at *U*_L_ is displayed as black line in Figure 2b. The difference between the equilibrium potential and the *U*_L_ is defined as theoretical overpotential (*η*_theo_), and the *η*_theo_ ≈ 0.7−0.63 V = 0.07 V for Pt–Hg_4_.

### 2.2 Kinetics and Volcano Plots for 2e^−^ ORR to H_2_O_2_

As noted earlier, the 2e^−^ ORR catalysts generally are also active for generation of water (thermodynamically favorable product). As the free diagram in Figure 2c, the key to avoid the 4e^−^ pathway is to prevent the O—O bond dissociation in the adsorbed *OOH. Thus, the catalysts with strong oxygen binding energies (favorable *O formation) are not suitable for 2e^−^ ORR, which limits the search to weak oxygen binding catalysts.^[^ ^5a^ ^]^ Additionally, suitable adsorption energy of *OOH on catalysts is also essential to achieve the high catalytic activity. For the 2e^−^ ORR catalytic process, the reaction pathway mainly consists of two steps, corresponding to the generation and removal of sole *OOH intermediate on the catalysts surface. Hence, the theoretical overpotential can be only determined as a function of the *OOH binding energy. A critical determining factor is whether the catalyst can dissociate the O—O bond, and the weak adsorption of intermediate will lead to high H_2_O_2_ selectivity but low activity. On the basis of scaling relations between different descriptors, corresponding to Δ*G*(*OOH) = Δ*G*(*OH) + 3.2 and Δ*G*(*O) = 2Δ*G*(*OH), the limiting potentials for 2e^−^ ORR can be expressed using *OH as a descriptor with the relationship as the Equations (4) and (5)

(4) $$U_{L1}=\;-\Delta G_{*\mathrm{OH}}+1.72$$

(5) $$U_{L2}=\Delta G_{*\mathrm{OH}}\;-0.32$$

Figure 2d presents the computed two‐electron volcano relation between the limiting potentials and Δ*G*(*OH) for the individual steps in Equation (4) (green line) and Equation (5) (purple line). The lowest limiting potential for the full catalytic reactions defines the overall limiting potential for the reaction, which is indicated by the purple and green solid lines. Because the catalytic process has only one *OOH intermediate, those two curves cross at the peak of the volcano, corresponding to the equilibrium potential at 0.70 V. As such, it is possible, in principle, to design one electrochemical catalyst with an ideal activity near the peak of volcano, where the binding strength between *OOH intermediate and catalyst is neither too weak nor too strong.^[^ ^10^ ^]^ As shown in Figure 2d, for catalysts with strong *OOH (or *OH) bonding energy lying on the left side of the two‐electron volcano (solid purple line), *OOH→H_2_O_2_ is potential limiting step and four‐electron ORR may dominates over the two‐electron pathway; On the other hand, in the case of weak *OOH binding lying on the right side of the volcano (solid green line), O_2_→*OOH is potential limiting step, which is expected to yield increased H_2_O_2_ selectivity but lower activity.^[^ ^11^ ^]^ As a result, the most promising catalyst with both high activity and selectivity toward H_2_O_2_ would be found at the apex of the 2e^−^ volcano plot.

## 3 Electrocatalysts and Factors Influencing 2e^−^ ORR Activity/Selectivity

For ideal 2e^−^ ORR catalysts, the adsorption of *OOH should be enhanced to achieve the high catalytic activity, while the adsorption of *O (the product of *OOH dissociation) should be reduced to obtain high selectivity.^[^ ^14^ ^]^ The electrocatalyst for the 2e^−^ reduction of oxygen should meet several criteria: high activity, operating with low catalyst loading, suitable conductivity, ideal mass transfer rate and high onset potentials/limiting diffusion current densities; high selectivity, ensuring high yields of H_2_O_2_ instead of H_2_O; high stability, enabling long‐term durability in various electrolytes. Earlier studies on 2e^−^ ORR to H_2_O_2_ generation mainly operated in alkaline solution, which can produce high alkalinity H_2_O_2_ solution for pulp and paper bleaching process. With the increasing application demand of H_2_O_2_, attractive electrochemical catalysts should also process well catalytic performance in acid and medium electrolyte.

**Figure 3:**
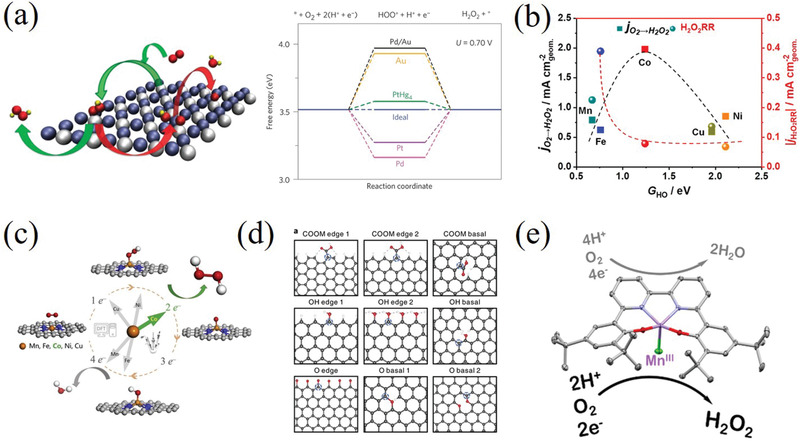
a) PdHg_4_ catalyst and free‐energy diagram of different catalysts for oxygen reduction to H_2_O_2_. Reproduced with permission.^[^ ^13^ ^]^ Copyright 2013, Springer Nature. b) Thermodynamic relations (volcano) lines for the two‐electron ORR of M–N–C catalysts. Reproduced with permission.^[^ ^16^ ^]^ Copyright 2019, American Chemical Society. c) Co–N–C SAC for H_2_O_2_ production. Reproduced with permission.^[^ ^17^ ^]^ Copyright 2020, Elsevier. d) Oxidized carbon catalyst with different oxygen functional groups for 2e^−^ ORR. Reproduced with permission.^[^ ^15a^ ^]^ Copyright 2018, Springer Nature. e) Molecular manganese complex with a bipyridine‐containing Schiff base ligand catalyst. Reproduced with permission.^[^ ^15b^ ^]^ Copyright 2018, American Chemical Society.

To date, numerous materials, such as noble metals/alloys, functional (O, N, F, S, B^−^) carbons, non‐noble transition metals, single‐atom catalysts and molecular complex, have been proposed to improve the catalytic performance of 2e^−^ ORR.^[^ ^8^, ^15^ ^]^ Rossmeisl and co‐workers reported Pt−Hg nanoparticles with ultrahigh catalytic performance for H_2_O_2_ production (**Figure** **3**a).^[^ ^13^ ^]^ The isolated Pt atoms surrounded by inert Hg atoms can effectively adsorb *OOH as well as decrease the O* binding energy strength, as reflected by the adsorption energies of *OOH/*O falling below the scaling line of single elemental metals. Several bimetallic alloys, such as Pd−Au and Pd−Hg, also show impressive mass activity and high selectivity (>95%) toward the synthesis of H_2_O_2_.^[^ ^9^, ^14^ ^]^ However, the scarcity of noble metals and toxicity of Hg significantly hinder their large‐scale applications. The state of d‐orbitals electrons in transition metals is also a crucial factor to affect the *OOH binding strength. Researchers screened out seven non‐noble transition metal catalysts toward two‐electron ORR with higher activity than the PtHg_4_ in acid media, by means of DFT computations.^[^ ^8^, ^12^ ^]^ The DFT results displays the predicted binding energy of *OH intermediate over Co−N−C catalyst is located near the top of the volcano accounting for favorable two‐electron ORR (Figure 3b).^[^ ^16^ ^]^ SAC is also a kind of attractive 2e^−^ ORR catalyst, due to its suitable binding energy between *OOH intermediate and high mass activity. As show in Figure 3c, Co SAC anchored in nitrogen‐doped graphene can generate H_2_O_2_ via 2e^−^ ORR pathway with high catalytic efficiency.^[^ ^17^ ^]^ The support in SAC generally is crucial for active sites atomic dispersion and oxygen species adsorption energy regulation.^[^ ^18^ ^]^ Carbon materials have widely served as low‐cost and highly active 2e^−^ ORR electrocatalysts to yield H_2_O_2_. As shown in Figure 3d, the surface oxidation approach can influence the structure of graphene catalyst, and the carbon atoms adjacent to oxygen functional groups (OFGs) (C–O–C and –COOH) are the active sites for 2e^−^ ORR to generate H_2_O_2_.^[^ ^15a^ ^]^ The molecular complex also processes attractive electrocatalytic performance for producing H_2_O_2_ under aqueous conditions. As displayed in Figure 3e, the molecular manganese complex with bipridine‐containing Schiff base ligand exhibit high H_2_O_2_ catalytic performance with 81 ± 4% Faradaic efficiency.^[^ ^15b^ ^]^ Generally, the electronic structure, physical/chemical structure, active sites, and operation conditions of those catalysts can be well regulated. Each type of electrocatalysts and factors affecting the electrochemical performance will be discussed in detail in the following parts.

### 3.1 Noble‐Metal Catalysts and Influence Factors

Noble‐metals are the most promising electrocatalysts for producing H_2_O_2_ via 2e^−^ ORR, which generally exhibit advantages of small overpotentials, ultrahigh H_2_O_2_ selectivity (up to ≈98%) as well as high stability. Numerous noble‐metal based catalysts, such as Au, Pd, and Pt, generally exhibit high catalytic performance for the electrochemical production of H_2_O_2_. To improve 2e^−^ catalytic performance, most studies are mainly focusing on the structure regulation of noble‐metal based catalysts, including electronic structure, particle size, types of support materials, defects and crystal grains, and functional groups. Besides the intrinsic electronic/crystalline structure of noble metals, several factors, such as alloying, particle size, mass loading, and interparticle distance, will influence the catalytic performance of the 2e^−^ ORR.

**Figure 4:**
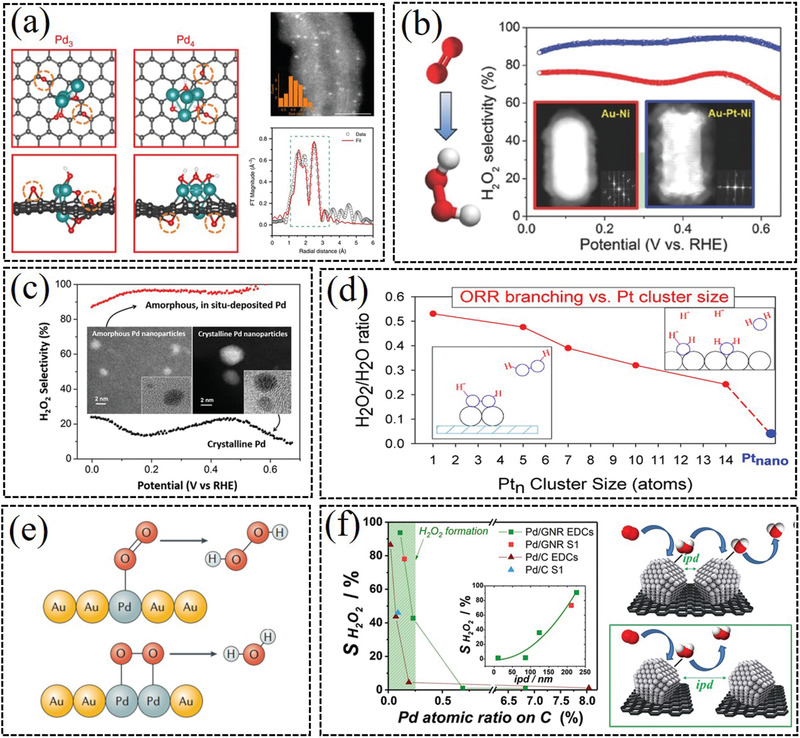
a) Promoting H_2_O_2_ production via 2e^−^ ORR by coordinating partially oxidized Pd and defect carbon. Reproduced with permission.^[^ ^20^ ^]^ Copyright 2020, Springer Nature. b) Au–Pt–Ni nanorods for high selectivity H_2_O_2_ production. Reproduced with permission.^[^ ^25^ ^]^ Copyright 2016, Wiley‐VCH. c) The H_2_O_2_ catalytic selectivity of Pd nanoparticles with amorphous and crystalline structure. Reproduced with permission.^[^ ^21^ ^]^ Copyright 2019, American Chemical Society. d) Size‐dependent properties of Pt*_n_*/ITO catalyst. Reproduced with permission.^[^ ^27^ ^]^ Copyright 2015, American Chemical Society. e) The “end on” and “side on” adsorption mode of oxygen to Pd–Au alloys. Reproduced with permission.^[^ ^29^ ^]^ Copyright 2019, Springer Nature. f) The catalytic performance of Pd/C with distinct interparticle distances. Reproduced with permission.^[^ ^22^ ^]^ Copyright 2018, American Chemical Society.

Various noble single metal catalysts, such as Au, Pd, and Pt, are investigated as the 2e^−^ ORR catalysts to produce H_2_O_2_. Previously, many Au based materials, such as Au/C, Au/Vulcan XC‐72R, and Au_25_(SC_12_H_25_)_18_, were studied as promising catalysts toward selective H_2_O_2_ generation, and the electrochemical reaction mainly occurred on Au (111) and Au (110) active crystalline planes.^[^ ^19^ ^]^ Chang et al. reported a Pd*^*δ*^* ^+^–OCNT electrocatalyst with nearly 100% selectivity toward H_2_O_2_ electrochemical production and a high mass activity (1.946 A mg^−1^ at 0.45 V) in acidic electrolyte (**Figure** **4**a).^[^ ^20^ ^]^ The X‐ray absorption fine structure characterization and DFT calculations demonstrate that the synergistic interaction between partially oxidized Pd clusters and oxygen‐functionalized CNT substrate is crucial for the high 2e^−^ ORR catalytic activity. Various other Pd/C catalysts displayed highly selectivity toward H_2_O_2_ production.^[^ ^21^ ^][^ ^22^ ^]^ Pt‐based catalysts also process high 2e^−^ ORR catalytic performance.^[^ ^23^ ^]^ For instance, Choi et al. reported that sulfur doped carbon (17 wt% S) could stabilize high loading of platinum (5 wt%), which exhibited high H_2_O_2_ selectivity (≈96%) via 2e^−^ ORR.^[^ ^23b^ ^]^

Noble metal alloys, with synergetic presence of two (or more) metals with different oxygen binding energies, generally exhibit excellent electrochemical catalytic activity for 2e^−^ H_2_O_2_ production. Siahrostami et al. firstly reported that Pt–Hg nanoparticles exhibited ultrahigh catalytic activity for generating H_2_O_2_, corresponding to selectivity up to 96% at 50 mV overpotential.^[^ ^13^ ^]^ The inert Hg atoms could surround isolated Pt atoms and adjust the binding energy between oxygen species and catalytic sites. Jirkovský et al. indicted that alloying Pd, Pt or Rh atoms on Au surface could enhance the H_2_O_2_ production relative to pure Au by DFT modeling.^[^ ^24^ ^]^ Recently, Zheng et al. reported an Au–Pt–Ni NRs ternary metal catalyst, which exhibited high catalytic selectivity of 95% toward H_2_O_2_ production between 0.45 and 0.55 V (vs RHE) (Figure 4b).^[^ ^25^ ^]^ Lots of noble metal alloys, such as Pt–Au–Cu nanowire and Pd–Au, also reported as high efficient 2e^−^ ORR catalysts for H_2_O_2_ production.^[^ ^26^ ^]^ Although the noble‐based catalysts generally process high 2e^−^ ORR catalytic performance in acid medium, the mass loading/mass activity of catalysts should be further optimized and the compatibility in alkaline/neutral electrolyte should be enhanced.

The crystalline/amorphous structure and particle size are the crucial factors to influence the catalytic performance of catalyst for 2e^−^ ORR. Kronawitter and co‐workers reported the amorphous Pd nanoparticles exhibited an ultrahigh H_2_O_2_ selectivity above 95%, which was significantly higher than that of crystalline Pd catalyst (Figure 4c).^[^ ^21^ ^]^ Various studies demonstrate that the particle size of noble‐metal clusters below ≈5 nm always promotes high catalytic performance for 2e^−^ ORR.^[^ ^9^, ^19^, ^21^, ^27^ ^]^ For instance, Anderson and co‐workers prepared size‐selected Pt*_n_* clusters on indium tin oxide (ITO) composite, and the maximized H_2_O_2_ selectivity was observed with the smallest Pt_1_ size species (Figure 4d).^[^ ^27^ ^]^ Jirkovský et al. discussed the effects particle size of Au nanoparticles on oxygen reduction selectivity for H_2_O_2_ production. The results showed that selectivity for H_2_O_2_ production was high for the catalyst with particle sizes below 6 nm.^[^ ^19b^ ^]^

The loading amount, distinct interparticle distances of noble metal active sites and oxygen species adsorption direction are also crucial to the electrocatalytic activity and selectivity of H_2_O_2_. It is demonstrated that a lower catalyst loading results in the sparse distribution of active sites and the dissociation of intermolecular O—O bond is hindered, which is beneficial to produce H_2_O_2_ via 2e^−^ ORR way.^[^ ^28^ ^]^ As shown in Figure 4e, on Pd–Au alloys with continuous Pd atom reactive sites, O_2_ is adsorbed with “side‐on” mode, which is conducive to O—O bond breakage and generation of H_2_O.^[^ ^29^ ^]^ However, isolated Pd atom catalytic sites prefer the “end‐on” binding mode for O_2,_ which hinders O—O bond breakage such that H_2_O_2_ can be preferentially formed. Choi et al. demonstrated that controlled coating of Pt catalysts with amorphous carbon layers could induce selective end‐on adsorption of O_2_ on the Pt surface, which exhibited significantly enhanced H_2_O_2_ production selectivity up to 41%.^[^ ^23a^ ^]^ Fortunato et al. suggested that the catalysts with different Pd loadings and distinct interparticle distances could influence the catalytic performance toward H_2_O_2_.^[^ ^22^ ^]^ As shown in Figure 4f, the generated H_2_O_2_ intermediate tends to diffuse into the electrolyte before further undergoing reduction to H_2_O, when the Pd interparticle distance is higher than 125 nm.

### 3.2 Carbon‐Based Catalysts and Influence Factors

Carbon based nanomaterials are recognized as promising 2e^−^ ORR electrocatalysts, due to the advantages of global abundance, low cost, high surface area, large pore volume, excellent stability, and good electrical conductivity.^[^ ^30^ ^]^ Some commercial porous carbon‐based materials, such as Vulcan XC‐72R,^[^ ^31^ ^]^ CMK‐3,^[^ ^32^ ^]^ carbon black,^[^ ^33^ ^]^ and Printex L6,^[^ ^34^ ^]^ exhibit high practical application prospects for H_2_O_2_ generation via electrochemical 2e^−^ ORR pathway. Various synthesized porous carbon materials, including graphite felt,^[^ ^35^ ^]^ nanotubes,^[^ ^36^ ^]^ hierarchically porous carbon,^[^ ^37^ ^]^ and redox modifiers (quinones and azo compounds),^[^ ^38^ ^]^ also display 2e^−^ ORR catalytic performance to yield H_2_O_2_ under acidic/neutral/alkaline conditions. The pure carbon catalysts generally process unideal catalytic performance, so structural reconstruction^[^ ^39^ ^]^ and heteroatom doping (O, N, F, S, P, and B)^[^ ^40^ ^]^ are useful strategies to boosting their 2e^−^ ORR catalytic performance. The catalytic performances of some carbon‐based catalysts are listed in **Table** **1**. Several crucial factors may influence the catalytic performances of catalysts, which include carbon defect, oxygen functional groups, nitrogen types, synergistic effect, and others (pH, applied potential, pore structure, and steric hindrance effect). In the following sections, we will discuss in detail the crucial factors that influence electrochemical performance of carbon‐based catalysts.

**Table 1:** The electrochemical catalytic performance of some carbon‐based catalysts

Electrocatalysts	Electrolytes	H_2_O_2_ [%]	*E*_onset_ vs RHE	*N*	Reference
CMK‐3	0.1 m KOH	90	≈0.80 V	2.2	^[^ ^41^ ^]^
rGO‐KOH	0.1 m KOH	≈100	≈0.8 V	≈2.0	^[^ ^42^ ^]^
Meso‐C	0.1 m KOH	≈100	≈0.7 V	≈2.0	^[^ ^43^ ^]^
Meso‐BMP‐800	0.1 m HClO_4_ 0.5 m H_2_SO_4_ + 0.05 m Na_2_SO_4_	65.2 95	≈0.60 V ≈0.70 V	≈2.7 2.1	^[^ ^39a^ ^]^
O‐doped CMK3	0.1 m K_2_SO_4_ 0.1 m KOH	78 90	≈0.45 V ≈0.8 V	≈2.4 ≈2.2	^[^ ^32^ ^]^
MNCs	0.5 m H_2_SO_4_	90	≈0.5 V	2.2	^[^ ^44^ ^]^
Porous carbon	0.1 m Na_2_SO_4_ + 0.5 m H_2_SO_4_	82.4	≈0.60 V	≈2.4	^[^ ^37^ ^]^
N‐doped graphitized carbon	0.1 m H_2_SO_4_ 0.1 m NaOH	80 40	0.4 V 0.71 V	2.4 3.2	^[^ ^40b^ ^]^
GOMC	0.1 m KOH	>90	0.81 V	≈2.2	^[^ ^45^ ^]^
NCA‐850	0.1 m KOH	≈100	≈0.8 V	≈2.0	^[^ ^46^ ^]^
O‐CNTs	0.1 m PBS 0.1 m KOH	≈85 ≈90	≈0.60 V ≈0.80 V	≈2.3 ≈2.2	^[^ ^15a^ ^]^
Oxo‐G	0.1 m KOH	>82	≈0.8 V	≈2.3	^[^ ^47^ ^]^
N‐rGO	0.1 m KOH	–	≈0.75 V	–	^[^ ^48^ ^]^
NF‐Cs	0.5 m H_2_SO_4_ 0.1 m KOH	85–88 89.6	0.7 V 0.8 V	2.3 2.2	^[^ ^49^ ^]^
N‐doped C	0.1 m KOH	93	0.88 V	≈2.1	^[^ ^50^ ^]^
N‐doped CMK3_800T	0.5 m H_2_SO_4_ 0.1 m K_2_SO_4_	98.5 89.8	0.49 V 0.52 V	2.0 2.1	^[^ ^51^ ^]^
N‐doped C	0.5 m H_2_SO_4_ 0.1 m K_2_SO_4_ 0.1 m KOH	85 85 95–98	≈0.40 V ≈0.45 V 0.78 V	2.3 2.3 2.1	^[^ ^39b^ ^]^
Graphitic N–C	0.1 m KOH	75	0.74 V	≈2.5	^[^ ^52^ ^]^
2,2′‐Dipyridylamine	0.5 m H_2_SO_4_	≈80	≈0.6 V	2.4	^[^ ^53^ ^]^
N,S‐MC	0.5 m H_2_SO_4_ 0.5 m KOH	76 75	≈0.32 V ≈0.56 V	2.5 2.5	^[^ ^40e^ ^]^
N‐MCs	0.1 m KOH	85	–	2.3	^[^ ^54^ ^]^
FPC	0.1 m Na_2_SO_4_ + 0.05 m H_2_SO_4_	93.6	≈0.3 V	≈2.1	^[^ ^14b^ ^]^
F‐mrGO(600)	0.1 m KOH	≈100	0.78 V	≈2.0	^[^ ^55^ ^]^

#### 3.2.1 Carbon Defects

**Figure 5:**
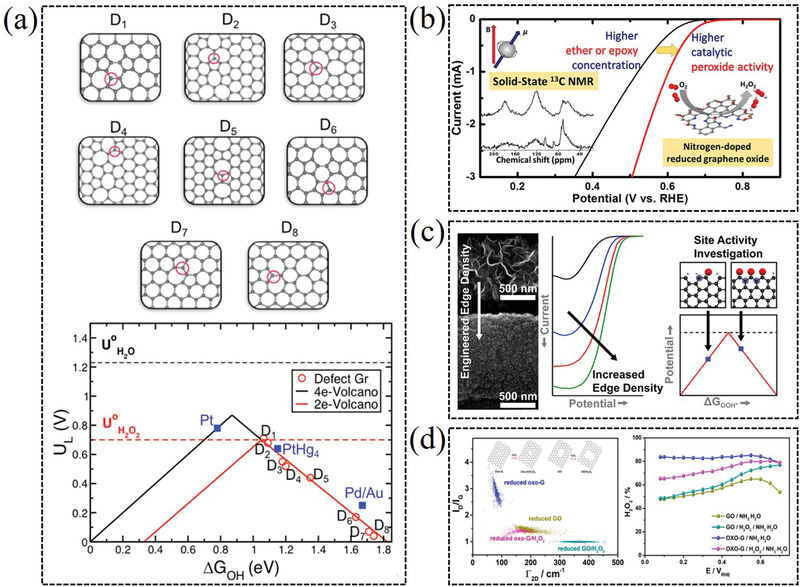
a) Different carbon defect type configurations examined in the DFT and two‐electron (red)/four‐electron (black) ORR‐related volcano plots. Reproduced with permission.^[^ ^43^ ^]^ Copyright 2018, American Chemical Society. b) Carbon defects with higher ether or epoxy for peroxide generation. Reproduced with permission.^[^ ^48^ ^]^ Copyright 2019, American Chemical Society. c) 3D out‐of‐plane graphene edge sites for highly selective 2e^−^ ORR electrocatalysis. Reproduced with permission.^[^ ^57^ ^]^ Copyright 2020, American Chemical Society. d) In‐plane carbon lattice‐defect of nitrogen‐doped graphene regulating electrocatalysis to H_2_O_2_ production. Reproduced with permission.^[^ ^47^ ^]^ Copyright 2019, American Chemical Society.

Substantial investigations have demonstrated that the carbon defects exhibit high 2e^−^ ORR catalytic activities, because the changes of geometrical and electronic structures can affect the binding strength of *OOH intermediate and the breaking energy of O—O bond.^[^ ^54^, ^56^ ^]^ For instance, Bao et al. prepared the microporous and mesoporous carbon catalysts, which exhibit a high onset potential near the thermodynamic equilibrium potential (0.7 V vs RHE) and a high selectivity of >70% for H_2_O_2_ (**Figure** **5**a).^[^ ^43^ ^]^ DFT calculation results demonstrate that various types of carbon defects in model graphene systems, including pentagon edges, single vacancies as well as double vacancies, are associated with catalytic performance for the 2e^−^ ORR. McCloskey et al. elucidated the influence of carbon defects to the 2e^−^ ORR catalytic performance (Figure 5b).^[^ ^48^ ^]^ They suggested that certain carbon defects associated with epoxy or ether groups have more pivotal role in promoting electrogeneration of H_2_O_2_ than other functionalities, such as nitrogen defects and carboxylic acid edge sites. Cohen‐Karni and co‐workers reported the nanowire‐templated 3D fuzzy graphene, with abundant carbonyl (C=O) and hydroxyl (C−OH) groups edge defectives, which exhibited notable 2e^−^ ORR catalytic efficiency (Figure 5c).^[^ ^57^ ^]^ They proposed that multiple functionalized configurations at both armchair and zigzag edges may achieve a local coordination environment for high efficiency 2e^−^ ORR. Eigler and co‐workers reported that the density of defects, including functionalization defects, carbon lattice defects, and nitrogen doping defects, were vital for regulating 2e^−^ ORR catalytic performance of the carbon catalysts (Figure 5d).^[^ ^47^ ^]^ Though defective carbons have shown good electrocatalytic performance toward H_2_O_2_, numerous defect‐site structures will be formed during preparation. Thus, it is a challenge for selectively tailoring synthetic method to induce the formation of the most active/selective active‐sites structure.

#### 3.2.2 Oxygen Functional Groups

**Figure 6:**
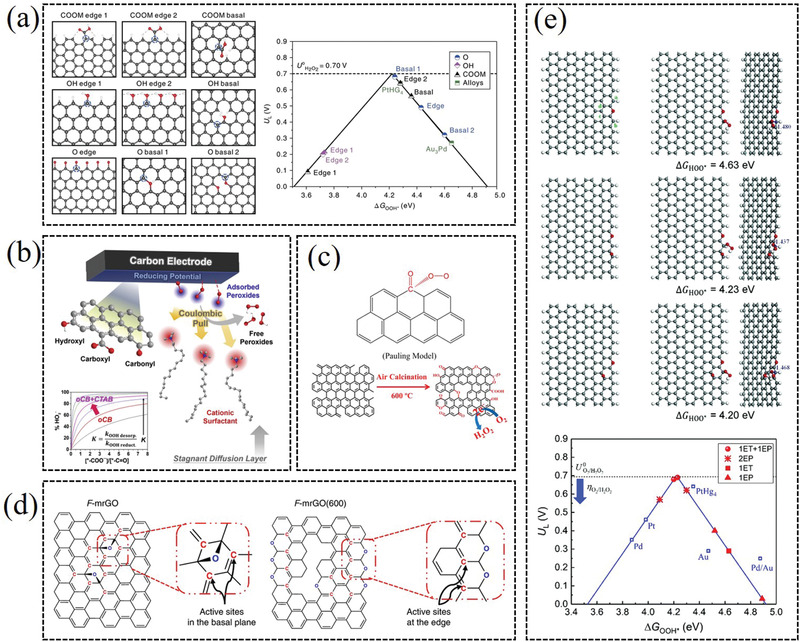
a) Oxidized carbon catalysts with various oxygen functional groups for 2e^−^ ORR. Reproduced with permission.^[^ ^15a^ ^]^ Copyright 2018, Springer Nature. b) In situ engineering of carbon catalyst with surface COO^−^ groups as main active sites and surface C=O groups for H_2_O_2_ selectivity. Reproduced with permission.^[^ ^59^ ^]^ Copyright 2020, Elsevier. c) Carbon black oxidized by air calcination for enhanced H_2_O_2_ generation. Reproduced with permission.^[^ ^33b^ ^]^ Copyright 2019, American Chemical Society. d) Reduced graphene oxide catalyst with sp^2^‐hybridized carbon near‐ring ether defects or sheet edges as active sites for H_2_O_2_ production. Reproduced with permission.^[^ ^55^ ^]^ Copyright 2018, Springer Nature. e) Mildly reduced graphene oxide catalyst with two types of oxygen functional group structures (2EP and 1ET + 1EP) as active sites for 2e^−^ ORR. Reproduced with permission.^[^ ^10^ ^]^ Copyright 2019, Royal Society of Chemistry.

Recently, OFGs, such as C=O, C—O and COOH, are identified to sufficiently enhance the catalytic activity and selectivity for H_2_O_2_ electrochemical synthesis via 2e^−^ ORR.^[^ ^58^ ^]^ Cui et al. observed that the oxidized carbon nanotubes (O‐CNTs) exhibited both high catalytic activity and selectivity (≈90%) for H_2_O_2_ production via electrochemical 2e^−^ ORR, and the catalytic properties were positively correlated with the oxygen content of the catalysts (**Figure** **6**a).^[^ ^15a^ ^]^ The DFT calculation indicates the carbon atoms adjacent to oxygen functional groups (–COOH and C–O–C) are the active sites for the 2e^−^ ORR. Recently, Guo et al. reported OFGs containing carbon‐based catalyst by in situ engineering with cationic surfactant, which delivered a high peroxide production with a sustainably high selectivity (96%) over 10 h (Figure 6b).^[^ ^59^ ^]^ The prepared catalyst contains abundant surface carboxylates (—COO^−^) and surface carbonyls (—C=O) functional groups. The surface COO^−^ groups are the main active sites with weak binding to surface peroxides, while surface C=O groups deteriorate selectivity by the strong binding with H_2_O_2_. Zhang et al. reported an air calcination method to improve the 2e^−^ ORR catalytic activity of commercial carbon black, in which OFGs exhibited main catalytic activity toward 2e^−^ ORR (Figure 6c).^[^ ^33b^ ^]^

Although the introducing of oxygen functional groups generally can improve the 2e^−^ ORR catalytic performance of catalysts, the accurate catalytic active sites are always controversial and crucial for further studies. McCloskey et al. demonstrated a mild thermal reduced graphene oxide (mrGO) electrocatalyst for efficient H_2_O_2_ production from O_2_ (Figure 6d).^[^ ^55^ ^]^ Spectroscopic structural characterization and DFT calculation provide strong evidence that the sp^2^‐hybridized carbon near‐ring ether defects along sheet edges are the most active sites for peroxide production. They proposed that the sp^2^‐hybridized carbon near the epoxy (EP) group on an unannealed mrGO basal plane and the ring ether (ET) defects along the annealed mrGO sheet edges are the most active sites for H_2_O_2_ production. Smith et al. examined the H_2_O_2_ formation activities of the active sites proposed by McCloskey, and found that their catalytic activities were actually very low by means of first‐principles calculations (Figure 6e).^[^ ^10^ ^]^ They systematically investigated the H_2_O_2_ formation activities of different oxygen functional group structures on mrGO based on experimental observations. They discovered that two types of oxygen functional group structures (2EP and 1ET + 1EP) exhibited comparable or even lower overpotentials (<0.10 V) for H_2_O_2_ formation compared with the state‐of‐the‐art PtHg_4_ electrocatalyst. Their theoretical results reveal that the graphene edge and the synergetic effects between different oxygen functional groups are essential for the superior performance of mrGO for H_2_O_2_ production.

#### 3.2.3 Nitrogen Types (Graphitic‐N, Pyridinic‐N, and Pyrrolic‐N)

**Figure 7:**
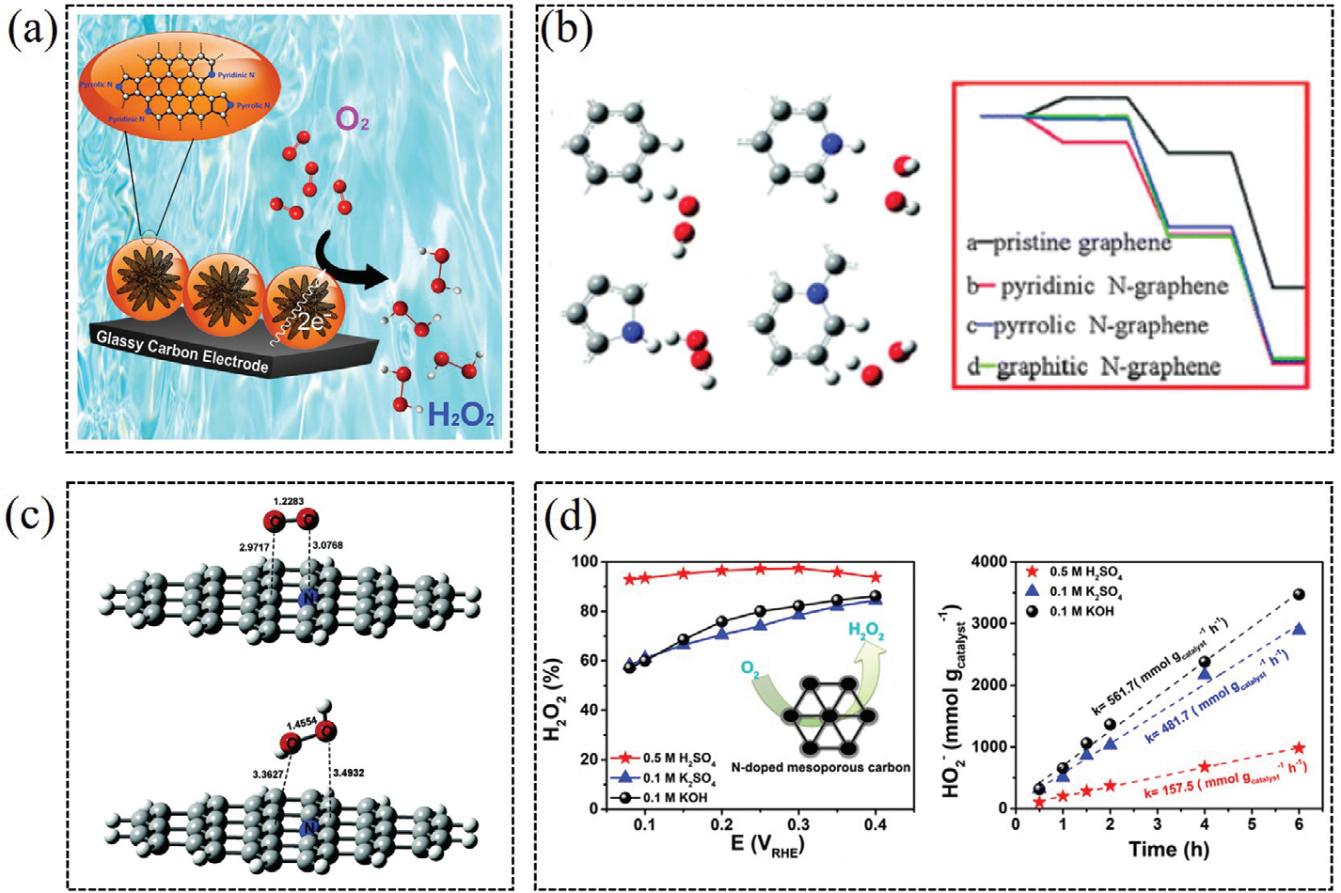
a) N‐doped graphitized carbon catalyst for highly production of H_2_O_2_. Reproduced with permission.^[^ ^40b^ ^]^ Copyright 2018, Elsevier. b) H_2_O_2_ production reactivity of nitrogen‐doped graphene within various carbon lattices. Reproduced with permission.^[^ ^61^ ^]^ Copyright 2013, Royal Society of Chemistry. c) 2e^−^ ORR catalytic performance of nitrogen‐doped graphite with graphite‐N as actives. Reproduced with permission.^[^ ^63^ ^]^ Copyright 2006, American Chemical Society. d) Nitrogen‐doped mesoporous carbon catalysts for H_2_O_2_ production. Reproduced with permission.^[^ ^39b^ ^]^ Copyright 2018, American Chemical Society.

The nitrogen dopants in the carbon‐based catalysts can significantly decrease the overpotential for the 2e^−^ ORR pathway, according to decreasing reaction Gibbs free‐energy and optimizing the binding energy of *OOH, respectively.^[^ ^60^ ^]^ For instance, Fornasiero et al. reported a N‐doped graphitized carbon nanohorns catalyst, which displayed high 2e^−^ ORR selectivity over a wide pH range (**Figure** **7**a).^[^ ^40b^ ^]^ The authors attribute the high H_2_O_2_ selectivity under acidic conditions to the effective protonation of the pyridine‐N, which reduces the catalytic center's ability to break the O—O bond. Wu et al. studied the microscopic relationship between the bonding configuration of various nitrogen‐doped graphene and the reactivity/mechanistic process toward H_2_O_2_ production via DFT calculation method (Figure 7b).^[^ ^61^ ^]^ They propose that the catalytic reactivity of various sites follows this order: pyridinic‐N graphene> pyrrolic‐N graphene> graphitic‐N graphene> pristine graphene. Except for the pyridinic‐N, the graphite‐N maybe is also feasible to H_2_O_2_ electroproduction via 2e^−^ ORR.^[^ ^62^ ^]^ For instance, Sidik et al. investigated the effect of graphite‐N sites in Ketjenblack to 2e^−^ ORR catalytic performance by an experimental and theoretical study. The quantum calculations demonstrate that the carbon radical sites formed adjacent to the graphite‐N are active for oxygen electroreduction to H_2_O_2_ (Figure 7c).^[^ ^63^ ^]^ Strasser et al. explored the 2e^−^ ORR electrocatalytic performance of a number of nitrogen‐doped mesoporous carbon catalysts, which achieved H_2_O_2_ selectivity of 95–98% in acidic solution (Figure 7d).^[^ ^39b^ ^]^ Nitrogen doping was found to sharply boost H_2_O_2_ activity and selectivity. Until now, most studies consent the pyridinic‐N and graphitic‐N are electroactive for H_2_O_2_ generation by adjusting the electronic structure of adjacent carbon sites, which is instructive for designing high efficiency 2e^−^ ORR nitrogen‐doped catalysts.

#### 3.2.4 Synergistic Effect

**Figure 8:**
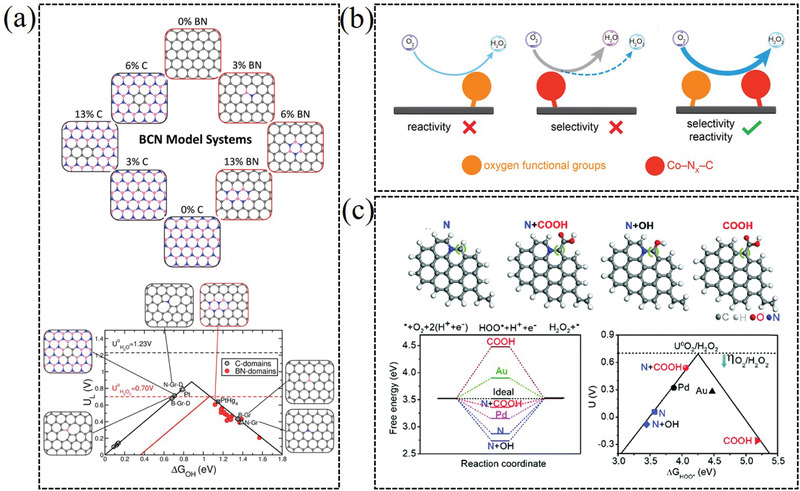
a) Designing boron nitride islands in carbon catalyst for efficient 2e^−^ ORR. Reproduced with permission.^[^ ^40a^ ^]^ Copyright 2018, American Chemical Society. b) Electrosynthesis of H_2_O_2_ synergistically catalyzed by atomic Co–N*_x_*–C sites and oxygen functional groups. Reproduced with permission.^[^ ^65^ ^]^ Copyright 2019, Wiley‐VCH. c) A COOH‐terminated nitrogen‐doped carbon aerogel as electrochemical catalyst for H_2_O_2_ generation. Reproduced with permission.^[^ ^46^ ^]^ Copyright 2019, Royal Society of Chemistry.

The synergetic effect between different functional groups and other catalytic active moieties is also crucial to adjust the catalytic activity of catalysts for H_2_O_2_ generation. Many catalysts generally consist of various active sites to maximally boosting their catalytic performance.^[^ ^64^ ^]^ For instance, Bao et al. reported a boron and nitride co‐doped carbon catalyst for efficiently electrochemical synthesis of H_2_O_2_ (**Figure** **8**a).^[^ ^40a^ ^]^ The *h*‐BN domains in graphitic structures provide higher activity and selectivity for the 2e^−^ ORR process in comparison to individual B or N doped structures. Furthermore, experimental and DFT results illustrate that the interface between *h*‐BN domains and graphene exhibits unique catalytic behavior and can preferentially drive the production of H_2_O_2_. Zhang and co‐workers reported that the Co−N*_x_*−C sites and oxygen functional groups, including C–O and –COOH species, contributed to the reactivity and selectivity for H_2_O_2_ electrogeneration, respectively (Figure 8b).^[^ ^65^ ^]^ The experimental results show that only atomic Co−N*_x_*−C sites can afford ideal ORR reactivity but poor selectivity for 2e^−^ ORR, while only abundant oxygen functional groups can provide high H_2_O_2_ selectivity and inferior ORR reactivity. Zhao et al. constructed a COOH‐terminated nitrogen‐doped carbon aerogel electrocatalyst, which exhibited a complete 100% selectivity to H_2_O_2_ with high yields and stability (Figure 8c).^[^ ^46^ ^]^ Both theoretical and experimental results indicate the real role of the N heteroatom coexisting with COOH groups in boosting the activation of oxygen for the 2e^−^ ORR. With the development of uncovering the catalytic active sites of catalysts, designing the catalysts with synergistic effect active sites maybe is potential strategy for high efficiency 2e^−^ ORR catalysts.

#### 3.2.5 Others (pH, Applied Potential, Pore Structure, and Steric Hindrance Effect)

**Figure 9:**
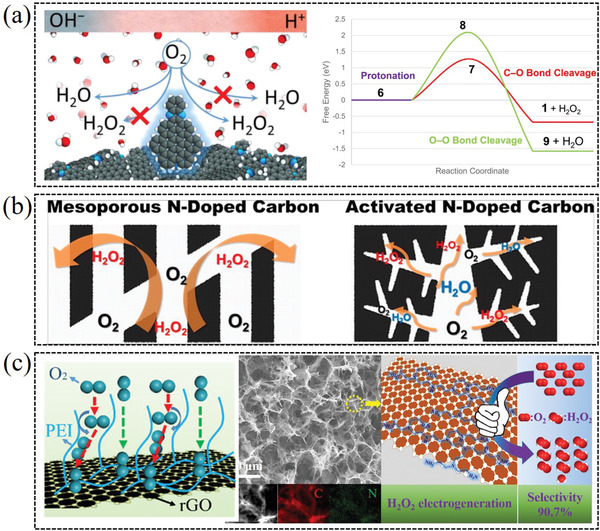
a) A model for the pH‐dependent selectivity of the ORR electro‐catalyzed by N‐doped graphitic carbon. Reproduced with permission.^[^ ^66^ ^]^ Copyright 2016, American Chemical Society. b) Mesoporous nitrogen‐doped carbon with highly selective 2e^−^ ORR performance than microporous catalyst. Reproduced with permission.^[^ ^44^ ^]^ Copyright 2014, American Chemical Society. c) Steric hindrance effect on PEI/rGO interfaces and the selectivity of H_2_O_2_ electrogeneration. Reproduced with permission.^[^ ^67^ ^]^ Copyright 2018, American Chemical Society.

For various 2e^−^ ORR catalysts, the pH environment also has an effect on the activity and selectivity for H_2_O_2_ production.^[^ ^39^, ^43^, ^51^, ^52^, ^66^ ^]^ As shown in **Figure** **9**a, Noffke et al. proposed a catalytic model based on interfacial solvation and dielectric constant to understand pH‐dependent selectivity for ORR.^[^ ^66^ ^]^ The synthesized N‐doped graphitic carbon catalyst displays the two‐electron ORR pathway in acid medium, while exhibits 4e^−^ ORR process in alkaline electrolyte. Iglesias et al. performed rotating ring disk electrode experiments under various pH conditions in order to confirm the selectivity of the catalytic process.^[^ ^40b^ ^]^ The results show that the lower pH with a higher proton concentration mainly leads to 2e^−^ reaction to H_2_O_2_, while the higher number of electrons of catalyst at alkaline pH is less selective toward H_2_O_2_ production. In addition, various studies demonstrate that both H_2_O_2_ selectivity trend and the number of transferred electrons are associated with the applied potential.^[^ ^51^, ^67^ ^]^ For instance, Xiao et al. investigated the influence of applied potential to the ORR selectivity using reduced graphene oxide aerogels catalysts. The effect of the applied potential on the ORR selectivity can be understood from the following two aspects. On one hand, the applied potential acts as a driving force for the catalytic reaction, which inevitably affects the reaction process. On the other hand, the adsorption of some spectator species (OH_ad_) are also affected by the applied potential, which in turn also affects the target reaction.^[^ ^67^ ^]^

The porous structures, including pore size and porosity, are crucial factors for catalytic performance of 2e^−^ ORR, mainly influencing the mass transport process in catalyst layer.^[^ ^14^, ^44^ ^]^ The presence of regular micropores may decrease the residence time of the H_2_O_2_ on the electrochemical catalysts, thus contributing to prevent further reduction of H_2_O_2_ to water. Park et al. prepared a series of mesoporous nitrogen‐doped carbon catalysts, exhibiting well‐ordered mesopores with diameters of 3.4–4.0 nm, which displayed high selectivity toward H_2_O_2_ over 90% (Figure 9b).^[^ ^44^ ^]^ Such high selectivity toward H_2_O_2_ is probably due to good mass transport of the mesoporous structure and abundant exposed active sites in the catalyst layer. Xiao et al. reported rGO/PEI aerogel catalyst with enhanced H_2_O_2_ electrogeneration selectivity, which was related to the steric hindrance effect (Figure 9c).^[^ ^67^ ^]^ The 3D porous structure of aerogels and the steric hindrance effect between PEI and rGO interface endow enhanced 2e^−^ catalytic selectivity (90.7%), production rate (106.4 mmol *g*_catalyst_^−1^ h^−1^) and durability for H_2_O_2_ electrogeneration.

### 3.3 Non‐Noble Transition Metal Catalysts

Non‐noble transition metal catalysts, such as transition metal oxide (TMO) and metal–nitrogen modified carbon (M–N–C, M = Mn, Fe, Co, Ni, Cu, etc.), are widely studied for electrocatalytic production of H_2_O_2_, due to their low cost and environmental compatibility. Nevertheless, the pristine TMO generally exhibits limited catalytic activities and insufficient durability, probably because of their intrinsic electronic structure, low conductivity, unideal mass transfer, and agglomeration. Loading metal oxides on conductive carbon supports is a promising strategy to overcome these limitations. For instance, using Vulcan XC 72 as carbon support, a series of TMO/C catalysts, such as CeO_2_/C,^[^ ^68^ ^]^ SnNi/C,^[^ ^69^ ^]^ TiO_2_–Au/C,^[^ ^70^ ^]^ V/C,^[^ ^71^ ^]^ ceria/C,^[^ ^72^ ^]^ and MnO_2_/C,^[^ ^58c^ ^]^ are investigated for the reduction of O_2_ to H_2_O_2_ with the high activity and selectivity. Printex L6 is another commercial carbon support to construct TMO/C catalysts for electrogeneration of H_2_O_2_, such as CeO_2_/C^[^ ^68b^ ^]^ and ZrO_2_/C.^[^ ^73^ ^]^ Additionally, some graphene supported metal oxide catalysts, such as *γ*‐Fe_2_O_3_@graphene,^[^ ^74^ ^]^ ZrO_2_‐rGO,^[^ ^75^ ^]^ and MnCO_3_/GO^[^ ^76^ ^]^ catalysts show high selectivity toward 2e^−^ ORR to H_2_O_2_ electrogeneration. Recently, some Zn and Co activated carbon catalysts were also used for electrogeneration of H_2_O_2_ via 2e^−^ ORR.^[^ ^77^ ^]^ The catalytic performances of some carbon‐based catalysts are listed in **Table** **2**. These studies show that lower quantities of metal on carbon supports are more active for H_2_O_2_ formation.

**Table 2:** The electrochemical performance of some non‐noble transition catalysts

Electrocatalysts	Electrolytes	H_2_O_2_ (%)	*E*_onset_ vs RHE	*N*	Reference
Nb_2_O_5_‐rGO	0.1 m K_2_SO_4_ 0.1 m NaOH	85.3 74.9	0.3 V 0.7 V	≈2.3 2.5	^[^ ^80^ ^]^
Co*_x_* O*_y_*/C	1 m NaOH	74	≈0.85 V	2.4	^[^ ^81^ ^]^
4% CeO_2_/C	1 m NaOH	88	≈0.75 V	≈2.2	^[^ ^68a^ ^]^
CeO_2_/C	1 m NaOH	44	≈0.75 V	3.1	^[^ ^68b^ ^]^
SnNi/C	1 m NaOH	88	≈0.7 V	≈2.2	^[^ ^69^ ^]^
CoS_2_	0.05 m H_2_SO_4_	≈70	≈0.7 V	2.6	^[^ ^82^ ^]^
Co–NC	0.1 m H_2_SO_4_	≈100	≈0.75 V	–	^[^ ^83^ ^]^
Fe_3_O_4_/Printex Fe_3_O_4_/graphene	1 m KOH	68 62	≈0.6 V	≈2.6 ≈2.8	^[^ ^84^ ^]^
Co–C	0.1 m HClO_4_	80±5	≈6.2 V	2.4	^[^ ^77d^ ^]^
Co−N−C	0.5 m H_2_SO_4_	80	≈0.78 V	2.4	^[^ ^16^ ^]^
Oxidized Co–N–C	0.1 m HClO_4_	> 85	–	–	^[^ ^85^ ^]^
Mn–N–C	0.1 m HClO_4_	> 98	≈0.7 V	≈2.0	^[^ ^78^ ^]^
Co–POC	0.1 m KOH	80	0.79 V	2.4	^[^ ^65^ ^]^

**Figure 10:**
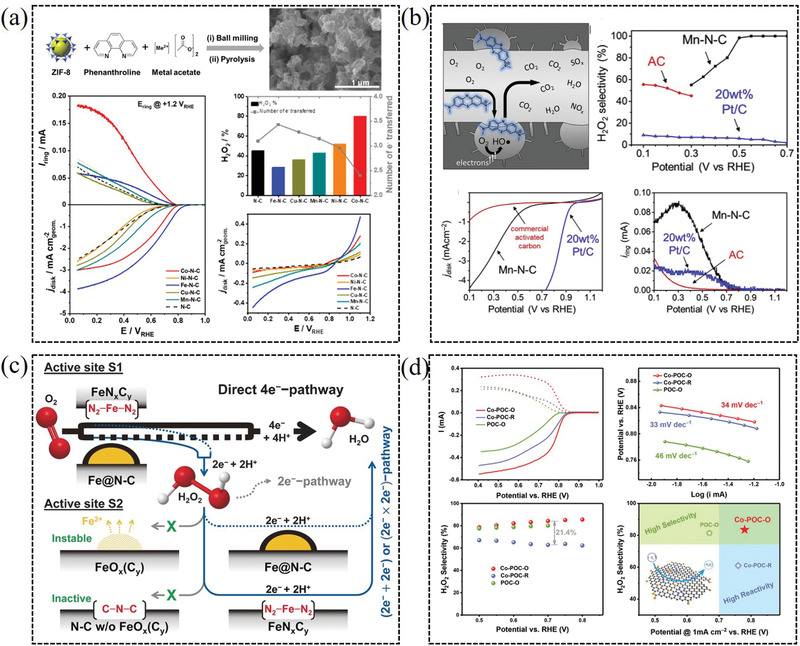
a) M–N–C catalysts on the catalytic activity and selectivity for hydrogen peroxide production. Reproduced with permission.^[^ ^16^ ^]^ Copyright 2019, American Chemical Society. b) Efficient H_2_O_2_ generation in highly porous manganese and nitrogen co‐doped carbon nanorods. Reproduced with permission.^[^ ^78^ ^]^ Copyright 2019, Elsevier. c) Investigation of the nature of active sites toward ORR in Fe–N–C catalysts. Reproduced with permission.^[^ ^79^ ^]^ Copyright 2017, Wiley‐VCH. d) Production of H_2_O_2_ synergistically catalyzed by atomic Co–N*_x_*–C sites and oxygen functional groups. Reproduced with permission.^[^ ^65^ ^]^ Copyright 2019, Wiley‐VCH.

Recently, the specific transition metals are introduced into nitrogen‐doped carbon frameworks with forming metal–nitrogen (M–N*_x_*) moieties to stabilize and activate metal cations.^[^ ^16^, ^65^ ^]^ The M–N*_x_* moieties generally are contributed to the high catalytic performance for 2e^−^ ORR. For instance, Strasser et al. combined computational and experimental efforts to uncover the trends in electrochemical H_2_O_2_ production over a series of M−N−C materials (**Figure** **10**a).^[^ ^16^ ^]^ The Co−N−C catalyst is uncovered with outstanding H_2_O_2_ productivity high selectivity (80%) in 0.5 m H_2_SO_4_. Additionally, porous manganese and nitrogen co‐doped carbon nanorods could catalyze reduction of oxygen in an acidic environment with >98% H_2_O_2_ selectivity (Figure 10b).^[^ ^78^ ^]^ Chio et al. investigated the active sites in Fe–N–C catalysts toward H_2_O_2_ production, which demonstrated that both FeN*_x_* C*_y_* moieties and Fe particles encapsulated in N‐doped carbon layers exhibited moderately catalytic activity toward 2e^−^ ORR (Figure 10c).^[^ ^79^ ^]^ Li et al. reported Co−N*_x_*−C and oxygen functional group co‐modified carbon electrocatalyst exhibited excellent catalytic performance for H_2_O_2_ generation, corresponding to a high selectivity over 80% in 0.10 m KOH (Figure 10d).^[^ ^65^ ^]^ For the non‐noble transition metal catalysts, optimizing the transition metal sites and functional carbon supports are essential to regulate their catalytic active sites, conductivity and mass transfer process, which can further improve the 2e^−^ ORR catalytic performance.

### 3.4 Single‐Atom Catalysts

In recent years, single‐atom catalysts (SACs) have received increasingly attentions for their particularly high activity and selectivity to produce H_2_O_2_ via 2e^−^ ORR pathway. One of the significant advantages of SACs is that it could offer near 100% utilization of metal atoms as active sites. More importantly, the underlying substrate can dramatically modify the electronic structure of supported single‐atom, thus altering the activity and selectivity of the active sites.^[^ ^17^, ^28^, ^86^ ^]^ Therefore, extensive investigations have been devoted into single‐atom catalysts to examine the possible structure–property correlation regarding activity and selectivity of single‐atom catalysts for H_2_O_2_ production.

**Figure 11:**
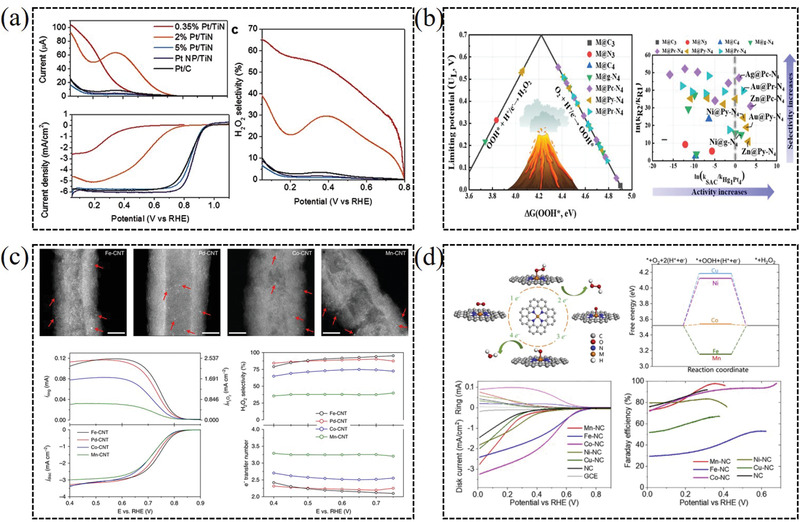
a) Single‐atomic platinum supported on TiN for selective 2e^−^ ORR. Reproduced with permission.^[^ ^28^ ^]^ Copyright 2015, Wiley‐VCH. b) DFT computations for representative experimentally achievable SACs toward 2e^−^ ORR. Reproduced with permission.^[^ ^12^ ^]^ Copyright 2019, American Chemical Society. c) Single‐atom Fe–C–O as an efficient H_2_O_2_ catalyst. Reproduced with permission.^[^ ^88^ ^]^ Copyright 2019, Springer Nature. d) Atomically dispersed cobalt anchored in nitrogen‐doped carbon as high efficiency electrocatalyst for H_2_O_2_ synthesis. Reproduced with permission.^[^ ^17^ ^]^ Copyright 2020, Elsevier.

As a sample, Lee and co‐workers predicted that Pt single‐atoms could be stabilized on N‐vacancy sites of titanium nitride support by the DFT calculations, and prepared Pt/TiN single‐atom catalyst with the high selectivity toward H_2_O_2_ (65%) generation.^[^ ^87^ ^]^ Unlike Pt nanoparticles, the Pt single‐atom catalyst can predominantly produce hydrogen peroxide in the electrochemical oxygen reduction with the highest mass activity reported so far (**Figure** **11**a).^[^ ^28^ ^]^ Tremendous efforts have done to comprehensively understand the underlying high selectivity/activity of single‐atom catalysts toward H_2_O_2_ production. Huang et al. simultaneously screened out seven single‐atom catalysts with higher activity and selectivity toward H_2_O_2_ production than the PtHg_4_ in acidic media by means of large‐scale DFT computations (Figure 11b).^[^ ^12^ ^]^ This machine‐learning method is very helpful for establishing the intrinsic structure−property correlations and accelerating the discovery of more efficient single‐atom catalysts via two‐electron ORR. Fe‐CNT SACs presents the maximum H_2_O_2_ selectivity of more than 95% in both alkaline and neutral electrolyte. The catalytic C and Fe active sites in Fe^−^C–O moieties are responsible for the H_2_O_2_ generation pathways by the DFT calculation (Figure 11c).^[^ ^88^ ^]^ Recently, Liu et al. systematically studied that the relation between the structure of transition metal (Mn, Fe, Co, Ni, and Cu) single‐atom catalysts anchored in nitrogen‐doped graphene and the catalytic performance for H_2_O_2_ synthesis. The results show that the Co single‐atom exhibits optimal d‐band center and can be operated as a highly active/selective catalyst for H_2_O_2_ synthesis (Figure 11d).^[^ ^17^ ^]^ In addition, Zhang et al. reported a hierarchical free‐standing single‐Co‐atom (with Co–N_4_ coordination) electrode to efficiently produce H_2_O_2_ via a 2e^−^ ORR pathway in acidic media.^[^ ^89^ ^]^ For the single‐atom 2e^−^ ORR catalysts, more work should be focused on improving the loading of single‐atoms and compatibility for various acid/alkaline/neutral electrolyte, which is vital for constructing membrane electrode assembly (MEA) and practical application. Some advanced technologies, such as machine‐learning method, characterization technique (electron microscope/synchrotron radiation) and DFT calculation, maybe are helpful to recognize the structure and catalytic active sites of the catalysts.

### 3.5 Molecular Complexes

Molecular complexes, including metal molecular complexes and nonmetal organic molecular complexes, are also widely investigated as heterogeneous two‐electron ORR catalysts in aqueous solutions at various pH values. Generally, the metal molecular complexes are composed of transition metal ion centers (mainly 3d transition metals, such as Fe, Co, Cu, Mn, and Ni) and organic macrocycles ligands (porphyrin, pyrazine, quinones and viologens‐type derivatives).^[^ ^90^ ^]^ Recently, various reports demonstrate that the nonmetal organic molecular complexes and noble‐metal molecular complexes also exhibit two‐electron ORR catalytic performance.

**Figure 12:**
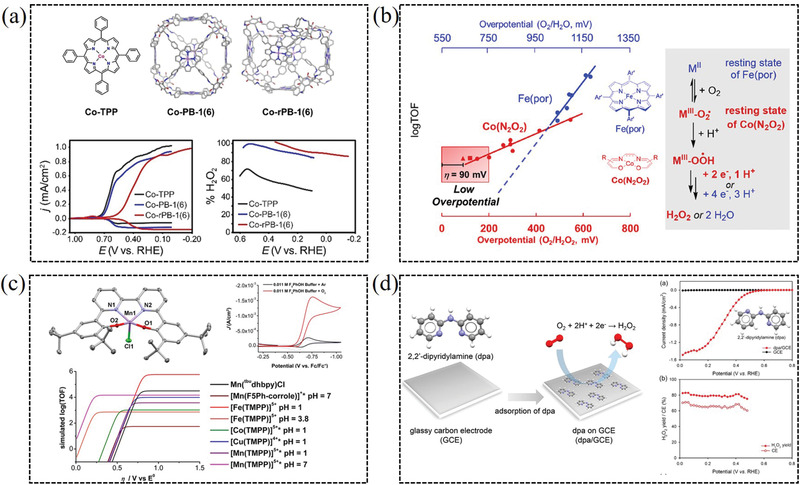
a) Supramolecular tuning enables selective electrosynthesis of H_2_O_2_ catalyzed by cobalt porphyrins. Reproduced with permission.^[^ ^95^ ^]^ Copyright 2020, Wiley‐VCH. b) N_2_O_2_‐ligated cobalt complexes for 2e^−^ ORR. Reproduced with permission.^[^ ^98^ ^]^ Copyright 2017, American Chemical Society. c) Molecular manganese complex with a bipyridine‐containing Schiff base ligand for selective H_2_O_2_ production. Reproduced with permission.^[^ ^15b^ ^]^ Copyright 2018, American Chemical Society. d) 2,2′‐dipyridylamine as heterogeneous organic molecular electrocatalyst for 2e^−^ ORR. Reproduced with permission.^[^ ^53^ ^]^ Copyright 2019, American Chemical Society.

The species of metal molecular complexes for 2e^−^ ORR catalysts mainly include iron, cobalt, and manganese complexes. In 1979, Bettelheim and Kuwana reported that the water soluble iron(III) tetra‐(4‐*N*‐methylpyridyl)porphyrin pentachloride (Fe^III^T4MPyP) could catalyze the electrochemical reduction of oxygen with an ultrahigh H_2_O_2_ yield of 95%.^[^ ^91^ ^]^ Thereafter, a series of Fe‐based molecular complexes are studied as 2e^−^ ORR catalysts, such as modification of ligands (Fe^III^TPPS)^[^ ^92^ ^]^ and synthetic metal centers (Cu(TPA) linked to iron porphyrin).^[^ ^93^ ^]^ Notably, cobalt molecular complexes, such as Co−porphyrins, Co−phthalocyanines, and Co‐macrobicyclic hexamine, generally exhibit desirable 2e^−^ electrochemical activity for producing H_2_O_2_.^[^ ^94^ ^]^ Smith et al. reported a cobalt tetraphenylporphyrin (Co‐TPP) supramolecular for direct electrosynthesis of H_2_O_2_ in neutral water. Site isolation of Co‐TPP active sites within a porous organic cage architecture enables high H_2_O_2_ selectivity (90–100%) and productivity (**Figure** **12**a).^[^ ^95^ ^]^ Additionally, some cobalt porphyrin polymers, such as pCoTAPP and Co(TCPP), are found to be efficient photo‐electrocatalysts for the photosynthesis of hydrogen peroxide via a 2e^−^ ORR pathway.^[^ ^94^, ^96^ ^]^ Extensive researches have been explored on the mechanism and activity of molecular Schiff base cobalt compounds for the selective electrocatalytic reduction of O_2_ to H_2_O_2_.^[^ ^97^ ^]^ For instance, S. Stahl reported one N_2_O_2_‐ligated cobalt complex catalyst using decamethylferrocene (Fc*) as the reductant and acetic acid as the proton source, which exhibited high selectivity toward H_2_O_2_ electroproduction (93−99%) (Figure 12b).^[^ ^98^ ^]^ With a similar catalytic mechanism of cobalt molecular derivatives, the Mn‐based molecular complexes, containing either porphyrins or porphyrin derivatives, can be also applied to electrocatalytically reduce O_2_ under aqueous conditions.^[^ ^99^ ^]^ Machan et al. reported a molecular manganese (III) complex, with a bipyridine‐containing Schiff base‐type ligand (Mn(^tbu^dhbpy)Cl), which is active for the electrocatalytic reduction of O_2_ to H_2_O_2_ with above 80% Faradaic efficiency. Mechanistic studies demonstrate that the catalytically active species have been generated through the interaction of the added proton donor and the parent Mn complex (Figure 12c). Stopped‐flow spectrochemical results demonstrate that the catalyst produces H_2_O_2_ by an proposed ECCEC (the abbreviation of reaction paths) mechanism, and less‐than‐quantitative selectivity is attributed to a thermal disproportionation reaction of H_2_O_2_.^[^ ^100^ ^]^

Besides the metal molecular complexes mentioned above, some metal‐free organic molecules have also shown catalytic activity for the two‐electron ORR. Yin et al. reported a heterogeneous organic molecular electrocatalyst, 2, 2′‐dipyridylamine, with a high H_2_O_2_ catalytic activity (onset potential of ≈0.60 V vs RHE) and selectivity (≈80%) in acidic aqueous electrolyte. In addition, the DFT study reveals a possible 2e^−^ ORR mechanism, in which the pyridyl‐ and amino‐N play as the anchoring sites for reaction intermediates (Figure 12d).^[^ ^53^ ^]^ Mitraka et al. reported that the conducting polymer PEDOT (poly(3,4‐ethylenedioxythiophene)) was an efficient catalyst for the reduction of O_2_ to H_2_O_2_ with Faraday efficiency close to 100%.^[^ ^101^ ^]^ Warczak et al. reported *N*,*N*′‐dimethyl perylenetetracarboxylic diimide (PTCDI) as an organic semiconductor catalyst for electrochemical generation of H_2_O_2_ in a pH range of 1–13, with a catalytic figure of merit up to 26 kg H_2_O_2_ per gram catalyst per hour.^[^ ^102^ ^]^ Peng et al. reported a 2D redox‐active cationic covalent triazine network as an electrocatalyst for generating H_2_O_2_, which exhibited high catalytic selectivity (≈85%) in a wide range of potentials (0.1–0.7 V vs RHE).^[^ ^103^ ^]^

## 4 Application of Electrogenerated H_2_O_2_ via 2e^−^ ORR

Hydrogen peroxide, as an important chemical, is widely used as an oxidant and disinfectant in human society. Considering the disadvantages of traditional anthraquinone oxidation–reduction and Huron–Dow methods to produce H_2_O_2_ (dilute alkaline H_2_O_2_ solution), electrochemical oxygen reduction method to produce H_2_O_2_ recently gained tremendous attention, which can be used in various fields. It is necessary that H_2_O_2_ could be synthesized in small amounts directly at the place of need and at the right point in time. Electrochemical synthesis is the most common alternative method for production of H_2_O_2_ via the 2e^−^ ORR.^[^ ^38^, ^68^ ^]^ This reaction makes the in situ H_2_O_2_ production from renewable power sources possible, and the scalability of electrochemical devices enables local and on‐demand H_2_O_2_ production, which can reduce the costs associated with storage and transportation.^[^ ^104^ ^]^ This electrochemical H_2_O_2_ production method has potential application in many fields, such as organic pollutants degradation, water treatment, bacteria killing and disinfection, and energy storage.

### 4.1 Organic Pollutants Degradation

Industrial wastewater often contains many toxic organic pollutants, such as organic dyes, organic drug and other harmful organic molecular, which can pose a considerable threat to human health and potential long‐term adverse effects on ecosystem.^[^ ^105^ ^]^ The Fenton method, based on the reaction between ferrous iron (Fe^2+^) and hydrogen peroxide (H_2_O_2_) to produce hydroxyl radicals (·OH), has been widely applied as advanced oxidation process. In the electro‐Fenton process, the electrogenerated H_2_O_2_ can be effectively utilized as Fenton reagent to produce ·OH, which can be further used for the degradation of organic pollutants.^[^ ^106^ ^]^ The state‐of‐the‐art electro‐Fenton process is mainly worked in acid media, as the optimum pH for the Fenton reactions is in the range of 2.8–3.0. Therefore, it is an effective method to remove various organic pollutants in wastewater.

#### 4.1.1 Organic Dyes

**Figure 13:**
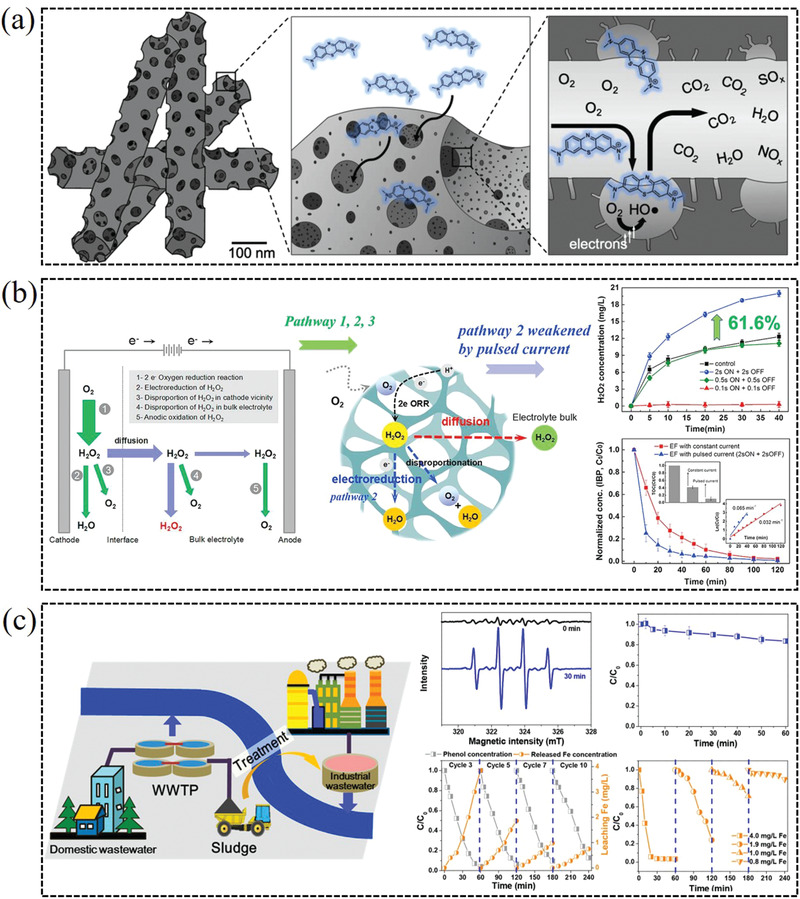
a) Organic dyes electrodegradation using porous manganese and nitrogen co‐doped carbon nanorods catalyst via 2e^−^ ORR. Reproduced with permission.^[^ ^78^ ^]^ Copyright 2019, Elsevier. b) Drastic enhancement of H_2_O_2_ electrogeneration by pulsed current for ibuprofen degradation. Reproduced with permission.^[^ ^113a^ ^]^ Copyright 2018, Elsevier. c) Electrochemically catalytic degradation of phenol with H_2_O_2_ in situ generated and activated by a municipal sludge‐derived catalyst. Reproduced with permission.^[^ ^115^ ^]^ Copyright 2018, American Chemical Society.

Amaranth is a typical representative of azo dyes. Producing H_2_O_2_ via 2e^−^ ORR under a neutral condition can be used in Fenton reaction for removal amaranth,^[^ ^107^ ^]^ which processes high removal rate. In the same way, methyl orange,^[^ ^35^, ^108^ ^]^ methylene blue (**Figure** **13**a),^[^ ^35^, ^78^ ^]^ acid blue 113 dye,^[^ ^109^ ^]^ acid orange,^[^ ^110^ ^]^ and rhodamine B^[^ ^33^, ^60^ ^]^ are also the common azo dye compounds existed in industrial wastewater. These dyes can be removed through the electrochemical reduction of O_2_ to H_2_O_2_ and in situ generation of hydroxyl‐free radicals. This method can efficiently degrade various organic pollutants and exhibit efficient total organic carbon removal in acidic or neutral condition.

#### 4.1.2 Organic Drug

Some pharmaceutical compounds, such as antibiotic, trace pharmaceutical compounds, tetracaine, and ibuprofen are frequently detected in the aquatic environment, which can also pose a considerable threat to human health and ecosystem. Therefore, they are usually chosen as the target contaminant. Tetracycline as a common antibiotic can be degraded using in situ generation of H_2_O_2_ and radicals.^[^ ^108^, ^111^ ^]^ Trace pharmaceutical compounds, such as carbamazepine, cimetidine and amoxicillin, can be totally degraded within 1.5–3 h.^[^ ^108b^ ^]^ Ridruejo et al. reported that the generated H_2_O_2_ by CoS_2_‐based catalyst in acidic medium could remove pharmaceutical tetracaine at 60–120 min by galvanostatic bulk electrolysis.^[^ ^112^ ^]^ As shown in Figure 13b, Ibuprofen can be also efficiently degraded by electro‐Fenton process.^[^ ^113^ ^]^

#### 4.1.3 Other Harmful Organic Molecules

Formaldehyde is harmful to the environment and human health. Therefore, treating industrial wastewater containing formaldehyde is of great importance. For instance, Wang et al. prepared a high‐performance electrocatalyst from commercial CMK3 for in situ H_2_O_2_ production, which exhibited high performance for reducing formaldehyde in wastewater.^[^ ^32^ ^]^ The results show that the removal efficiency of formaldehyde (initial concentration of 14.0 mg L^−1^) at different cell potentials are just for 30 min, corresponding to the highest efficiency at 2.7 V (up to 95%). Additionally, this method can markedly decrease the other harmful organics in wastewater, such as Phenol,^[^ ^35a^ ^]^ 4‐chlorophenol,^[^ ^114^ ^]^ bisphenol A, dimethyl phthalate, and perfluorooctanoate, which are chosen as model contaminants due to their environmental persistence, bioaccumulation, and potential toxicity (Figure 13c).^[^ ^115^ ^]^ Those model contaminants can be efficiently removed through the heterogeneous electro‐Fenton process for the selective two‐electron ORR to intermediate H_2_O_2_, and the efficient total organic carbon removal can reach to 88–99% in short time.^[^ ^106^ ^]^ In addition, organophosphorus compounds, such as glyphosate and N (phosphonomethyl) glycine, have been extensively used as herbicide and classified as “probably carcinogenic in humans,” which are also efficiently degraded using the same methods.^[^ ^116^ ^]^ Therefore, in situ electrogeneration of H_2_O_2_ through 2e^−^ ORR represents a potentially greener route for organic pollutants treatment in wastewater.

### 4.2 Water Treatment

**Figure 14:**
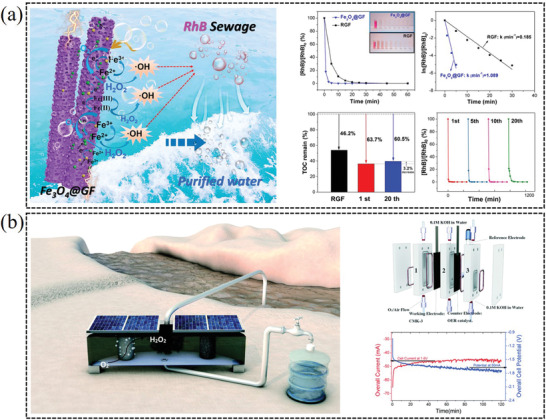
a) Fe_3_O_4_@GF catalyst combined with electro‐Fenton process for achieving swift water purification. Reproduced with permission.^[^ ^118^ ^]^ Copyright 2019, American Chemical Society. b) CMK‐3 based carbon catalyst for electrochemical H_2_O_2_ generation and water purification. Reproduced with permission.^[^ ^41^ ^]^ Copyright 2017, Royal Society of Chemistry.

The remarkable oxidation property of hydrogen peroxide allows it to oxidize various pollutants in waste water.^[^ ^117^ ^]^ Therefore, small‐scale decentralized electrochemical production of H_2_O_2_ via a 2e^−^ ORR offers unique opportunity for sanitization applications and the purification of drinking water. For instance, Lian et al. report that the Fe_3_O_4_@GF (graphite felt) and in situ formed PGF (porous graphite felt) are attractive self‐standing carbon materials for electrosynthesis of H_2_O_2_ and wastewater treatment, which simultaneously provide iron source toward electro‐Fenton process for hydroxyl radicals (•OH) production. Furthermore, the degradation performance of PGF does not significantly decay even after 20 cycles of the repeated use, which provides the possibility to achieve swift water purification (**Figure** **14**a).^[^ ^118^ ^]^ In addition, Li et al. reported that the in situ generated H_2_O_2_ in cathode combing with Fe^2+^ in anode could produce •OH via Fenton reaction to remove arsenite in solution, by efficiently oxidizing As(III) into As(V) and forming Fe(III)‐As(V) precipitates, which is practically meaningful to apply in the degradation and removal of organic pollutants in water.^[^ ^119^ ^]^ As another example, Chen et al. reported that H_2_O_2_ synthesis using carbon catalysts in reaction bath could be directly applied in bleaching and the treatment of acidic waste streams (Figure 14b).^[^ ^41^ ^]^ Additionally, electrochemical synthesis of H_2_O_2_ in alkaline media is highly necessary. The alkaline H_2_O_2_ solution is widely used for bleaching and brightening in the pulp and paper industry as well as in many other areas.^[^ ^8^, ^77^ ^]^

### 4.3 Bacteria Killing and Disinfection

**Figure 15:**
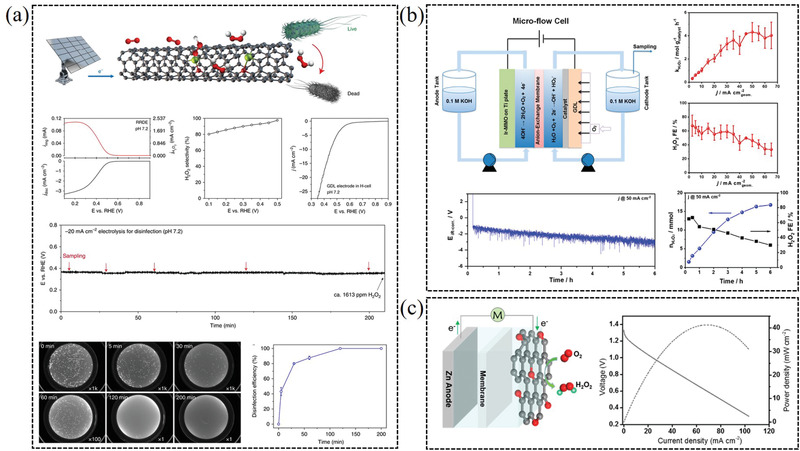
a) Fe–C–O as an efficient H_2_O_2_ catalyst for antibacteria and water disinfection. Reproduced with permission.^[^ ^88^ ^]^ Copyright 2019, Springer Nature. b) Microflow cell setup and H_2_O_2_ production rate at respective current densities on a Co–N–C electrode. Reproduced with permission.^[^ ^16^ ^]^ Copyright 2019, American Chemical Society. c) Oxygenated carbon electrocatalyst highly efficient production of electrical energy and H_2_O_2_ through a Zn–Air Battery. Reproduced with permission.^[^ ^122^ ^]^ Copyright 2018, American Chemical Society.

Hydrogen peroxide can be used in bacteria killing, which is one promising field in the delocalized or green‐route water disinfection.^[^ ^120^ ^]^ Jiang et al. reported a low‐cost Fe‐CNT catalyst for highly efficient H_2_O_2_ generation and performed a prototype experiment to test the water disinfection effectiveness of the catalysts (**Figure** **15**a).^[^ ^88^ ^]^ The results demonstrate that Fe‐CNT has a rapid disinfection efficiency for *Escherichia coli*, delivering a 43% bacteria inactivation in 5 min and more than 99.9999% in 120 min with no recovery observed. In addition, Wang et al. reported GO*_x_*/MnCO_3_ catalyst can be used for in situ electrochemical synthesis of H_2_O_2_ via the 2e^−^ pathway, which can be onsite decomposed to form •OH radicals in neutral media to further kill bacteria.^[^ ^76^ ^]^ Therefore, in situ electrochemical synthesis of H_2_O_2_ via 2e^−^ ORR can provide an effective and environmentally friendly electrochemical approach for killing bacteria and disinfection applications.

### 4.4 Energy Storage

Hydrogen peroxide as an environmentally benign energy carrier can be produced by the electrocatalytic two‐electron ORR (O_2_ from abundant in air), which can be used to generate electricity through the setup of H_2_O_2_ fuel cells.^[^ ^121^ ^]^ Strasser et al. reported a Co–N–C catalyst with high 2e^−^ catalytic performance during large‐scale H_2_O_2_ production at high current densities in 0.1 m KOH (Figure 15b).^[^ ^16^ ^]^ When evaluated in a commercial microflow cell (MFC), the Co–N–C catalyst exhibits an unprecedented production rate of more than 4 mol peroxide *g*_catalyst_^−1^ h^−1^ at a current density of 50 mA cm^−2^. Chen and co‐workers demonstrated the cogeneration of electrical energy and H_2_O_2_ through a liquid Zn−air battery based on the oxygenated carbon black (Figure 15c), which could convert chemical energy into electrical energy for energy storage.^[^ ^122^ ^]^ In addition, hydrogen peroxide is also utilized as an alternative liquid oxidant in place of gaseous O_2_.^[^ ^121^ ^]^ Day et al. developed a successful photosynthetic process, focusing on the reduction of oxygen to H_2_O_2_ as a means of chemical energy storage, which could convert light energy into chemical energy for energy storage.^[^ ^96^ ^]^

## 5 Summary and Outlook

H_2_O_2_ is a versatile and nontoxic commodity chemical, which is widely used in various fields. Electrochemical oxygen reduction via 2e^−^ pathway, instead of the industrial energy‐intensive anthraquinone process and direct synthesis method, becomes increasingly important as an alternative/green method for H_2_O_2_ generation. Here, we summarized the development of 2e^−^ ORR electrochemical catalysts in recent years, in aspects of mechanism exploration, types of high‐performance catalysts, factors to influence catalytic performance and potential applications. Until now, a diverse range of electrochemical catalysts are investigated for the electrochemical synthesis of H_2_O_2_, such as noble metal/alloys, carbon‐based materials, non‐noble transition metals, single‐atom catalysts and molecular complexes. We have elucidated the factors that control the catalysis of electrochemical H_2_O_2_ production, such as electronic structure, carbon defect, functional groups (O, N, B, S, F etc.), synergistic effect, pH, pore structure, and steric hindrance effect. Additionally, the electrochemical synthesis of H_2_O_2_ exhibits attractive potential applications, containing wastewater treatment, organics degradation, disinfection, and energy storage.

Although intensive researches have been done in recent years, the grant challenges still exist for efficiently producing H_2_O_2_ via 2e^−^ ORR and exploring suitable in situ application of generated H_2_O_2_. Electrocatalyst is always the most critical factor to influence the generation efficiency of H_2_O_2_. The current 2e^−^ ORR catalysts generally exhibit lower catalytic efficiency, obscure catalytic sites, and inferior stability in various electrolytes. An ideal catalyst for two‐electron ORR to produce H_2_O_2_ means that the adsorption of OOH* should be neither too strong nor too weak. And the selectivity of the catalysts is related to its ability to split the O—O bond. For the practical application, the 2e^−^ ORR catalysts also should process the outstanding mass activity, conductivity and mass transfer performance. Additionally, the application of the electrochemical generation of H_2_O_2_ via 2e^−^ ORR can be deeply developed with various in situ devices, which is attractive in many scientific/industrial fields. In the further, more research maybe should be focused on the following aspects to further developing desirable 2e^−^ ORR electrocatalyst and promoting the wide application of 2e^−^ ORR.

i) Understanding mechanism of 2e^−^ ORR to H_2_O_2_

Exploring the catalytic mechanism is crucial and challengeable for developing high efficiency 2e^−^ ORR catalysts. Some crucial technologies, including DFT calculation, high‐throughput screening studies, machine learning method, and advanced characterization techniques (electron microscope/synchrotron radiation), can help us recognize the catalytic sites and catalytic mechanism. Among them, the DFT calculation is extremely useful for describe the key adsorption energies and reaction energy barriers. Nørskov et al. firstly developed the DFT calculation method using CHE model to describe the free energies of the intermediates and solvated protons/electrons on catalyst surface at a given potential. A common DFT calculation theme is based on that the 2D surfaces of catalysts (such as specific metal crystalline face, modified graphene, MN_4_ moieties in graphene, ultrathin 2D metal oxides, boron nitride, etc.) interact with transition intermediates through oxygen atom. Generally, the well scaling relation between *OOH and *OH adsorption energies limits the 2e^−^ ORR catalysts with both high catalytic activity and selectivity. To obtain 2e^−^ ORR catalysts with intrinsic high catalytic efficiency, we need to explore catalysts that break the scaling between *OOH and *OH adsorption energies. This will require active sites that bind *OOH and *OH differently. One possible strategy is introducing multifunctional active sites, which exhibit proper combination of binding sites. More factors should be considered in the further DFT calculation, including bifunctionality, interfacial sites, surface functionalization, confinement, electrolyte engineering etc. Some advanced DFT methods, such as post Hartree–Fock methods, cluster/DFT embedding schemes and hybrid functional, will be necessary to model those multifunctional catalysts in the future. Additionally, the high‐throughput computational method is promising to discover new materials. The developing of advanced characterizations and analysis technologies, such as electron microscope/synchrotron radiation/nuclear magnetic resonance/machine learning method, are also vital to understand the catalytic sites and catalytic mechanism of 2e^−^ ORR.

ii) Factors that effect on achieving the desired ORR selectivity for H_2_O_2_

We have summarized the factors that influence the 2e^−^ ORR catalytic performance, including electronic structure, carbon defect, oxygen (N, B, etc.) functional groups, synergistic effect, pH, pore structure, and steric hindrance effect. Those factors are related to different catalytic active sites and further influence the catalytic performance. Through lots of catalytic mechanism are speculated, the universal relationships between the catalytic performance and structure of catalysts are still unclear. Meanwhile, insufficient understanding of which reaction steps are catalyzed by what sites, limits their progress. Therefore, more experimental and theoretical works are needed to elucidate the general relationship between 2e^−^ catalytic performance and catalytic sites, catalysts’ structure and the aforementioned factors.

**Figure 16:**
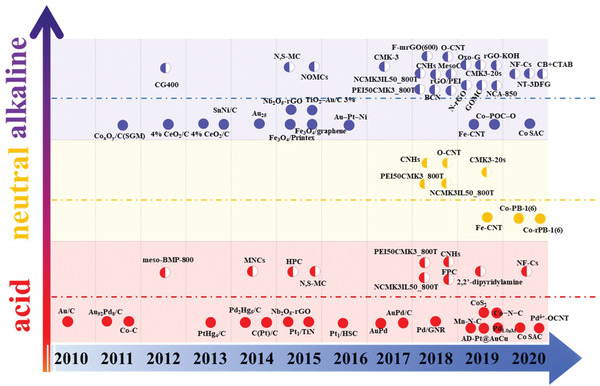
The diagram of summary some representative catalysts for H_2_O_2_ production through 2e^−^ oxygen electrochemistry in recent years (solid circle: metal catalyst; semisolid circle: nonmetal catalyst).

The recent research tendency of some representative 2e^−^ ORR catalysts in acidic/alkaline/neutral electrolyte are summarized in **Figure** **16**. It is noteworthy that most researches focus on the electrochemical generation of H_2_O_2_ in acidic and alkaline electrolyte, while the studies in neutral electrolyte are limited. In view of practical application, H_2_O_2_ produced in acidic medium can be usually used for oxidation purpose in organic synthesis, wastewater treatment or energy storage and conversion, and H_2_O_2_ generated in alkaline solution could be used for pulp and paper bleaching. Generally, the H_2_O_2_ produced in acidic and alkaline medium has limited practical application due the influence of pH. Thus, H_2_O_2_ produced in neutral aqueous should be studied intensively, which is thought to be the most useful and flexible form in their practical applications.

iii) The design of efficient catalysts

Plentiful catalysts have been studied for electrochemical generation H_2_O_2_ via 2e^−^ ORR pathway. For the practical application, the 2e^−^ ORR catalysts also should process outstanding catalytic activity/selectivity/stability, mass activity, conductivity, mass transfer performance and low cost. It still processes huge challenges to further develop high performance 2e^−^ ORR catalysts as follows.

Noble‐metal/alloys catalysts process high catalytic efficiency, while the high price of catalyst hinders their large‐scale application. More work should be done to optimize their mass loading/mass activity, and the compatibility in alkaline/neutral electrolyte in the future. Single‐atom catalysts generally process proper catalytic selectivity and the highest atomic catalytic performance. However, the loading of single‐atoms is generally low, and it still lacks an efficient route for synthesizing single‐atom catalysts with high yield and stability. Carbon based materials also display suitable 2e^−^ ORR catalytic performance and attractive application prospect, due to their high conductivity/stability, excellent porosity for mass transfer and low‐cost. More works should focus on improving their catalytic efficiency and exploring the catalytic sites/mechanism via conjunction of experimental/theoretical calculation. Structural reconstruction and heteroatom doping (especially oxygen or nitrogen doping) of carbon catalysts are useful strategies to boost their 2e^−^ ORR catalytic performance. Continuous studied are encouraged to screen out other useful nonmetallic heteroatoms (co‐)doping carbon materials. Non‐noble metal and molecular complex catalysts are widely studied for electrochemical generation of via 2e^−^ ORR. Optimizing the transition metal sites and functional supports/coordination ligands are crucial to regulate their catalytic active sites, conductivity, and mass transfer process. Additionally, more advanced technologies, such as high‐throughput screening studies, post DFT calculation, advanced characterization techniques, and machine learning method, can be used to further screen out highly efficient catalysts and uncover the catalytic sites/catalytic mechanism. It is most likely that in the next years a proliferation of new/cheap/advanced catalysts may exhibit high electrocatalytic activity/selectivity and stability toward 2e^−^ ORR, where the complex mechanistic features can be mastered at the nanoscale.

iv) Application of producing H_2_O_2_ with 2e^−^ ORR

H_2_O_2_ as renewable and clean energy source is widely used as a versatile and nontoxic commodity chemical. Environmental concerns are set to increase the demand for H_2_O_2_ over the coming year. Electrogeneration of H_2_O_2_ via 2e^−^ ORR, has become an emerging research field because of its flexibility and sustainability. It can be widely applied in many fields, such as organic pollutants degradation, water treatment, bacteria killing and disinfection, and energy storage. However, there are still many environmental fields and energy fields remaining to be further developed and utilized, such as protection against marine fouling organisms and energy storage/conversion. There is the lack of reported information on these above application fields. In addition, most applications are highly dependent on the electro‐Fenton process by using H_2_O_2_ to form ·OH. Therefore, developing novel application fields of H_2_O_2_ electrogeneration is necessary, which will not only trigger industrial interest for the development of new preparative schemes in conjunction with sustainability, but also realize on‐site generation of H_2_O_2_ from 2e^−^ ORR for real‐time field use. Future scrutiny is desired to improve the catalytic activity of catalysts in various acid/alkaline/neutral electrolytes. The development of on‐site setups combined with electrochemical generation of H_2_O_2_ is also crucial to promote its practical application.

## Conflict of Interest

The authors declare no conflict of interest.
